# A meta-analysis of infection rates of *Schistosoma japonicum* in sentinel mice associated with infectious waters in mainland China over last 40 years

**DOI:** 10.1371/journal.pntd.0007475

**Published:** 2019-06-07

**Authors:** Chen Qiu, Hui-Ying Zou, Yao Deng, You-sheng Liang, Da-Bing Lu

**Affiliations:** 1 Department of Epidemiology and Statistics, School of Public Health, Soochow University, Suzhou, China; 2 Jiangsu Key Laboratory of Preventive and Translational Medicine for Geriatric Diseases, School of Public Health, Soochow University, Suzhou, China; 3 Key Laboratory of National Health and Family Planning Commission on Parasitic Disease Control and Prevention, Jiangsu Provincial Key Laboratory on Parasite and Vector Control Technology, Jiangsu Institute of Parasitic Diseases, Wuxi, Jiangsu Province, China; 4 Jiangsu Provincial Key Laboratory on Parasites and Vector Control Technology, Wuxi, China; 5 Jiangsu Institute of Parasitic Diseases, Wuxi, China; Sichuan University West China Hospital, CHINA

## Abstract

**Background:**

Schistosomiasis japonica is a zoonotic parasitic disease. After nearly 70 years of control efforts in China, Schistosomiasis transmission has been reduced to a much lower level. The absence or near absence of infections in humans or livestock, based on traditional fecal and serological tests, has made the targets and priorities of future control efforts difficult to determine. However, detection of schistosome cercariae in waters using sentinel mice could be an alternative way of identifying remaining foci of infection, or even serve as a tool for evaluation of control efficacy. This method has been employed in China over last forty years. We therefore performed a meta-analysis of the relevant research to investigate if infections in sentinel mice mirror the ongoing trend of schistosomiasis transmission in China.

**Methods:**

We conducted a meta-analysis of studies reporting infection rates of *S*. *japonicum* in sentinel mice in China before Sep 1, 2018 in accordance with the PRISMA guidelines. We retrieved all relative studies based on five databases (CNKI, WanFang, VIP, PubMed and Web of Science) and the reference lists of resulting articles. For each individual study, the infection rate in sentinel mice is presented together with its 95% confidence interval (CI). Point estimates of the overall infection rates and their 95% CIs were calculated. Subgroup analyses were performed according to study periods, seasons or regions.

**Results:**

We identified 90 articles, including 290 studies covering eight endemic provinces. The overall rate in sentinel mice was 12.31% (95% CI: 10.14–14.65%) from 1980 to 2018. The value of 3.66% (95% CI: 2.62–4.85%) estimated in 2004 to 2018 was significantly lower than in 1980 to 2003 (22.96%, 95% CI: 19.25–26.89%). The estimate was significantly higher in the middle and lower reaches than in the upper reaches of the Yangtze River. The highest estimates were obtained in Hunan (30.11%, 95% CI: 25.64–34.77%) followed by Anhui (26.34%, 95% CI: 12.88–42.44%) and then Jiangxi (13.73%, 95% CI: 6.71–22.56%). Unlike the other provinces in the middle and lower reaches, no significant reduction was seen in Hubei after 2003. Even in Hubei two studies carried out after 2014 reported infections in sentinel mice, although no infected snails were reported across the province. Infections were most found in April (17.40%, 95% CI: 1.13–45.49%), July (24.98%, 95% CI: 15.64–35.62%) and October (17.08%, 95% CI 5.94–32.05%). High degrees of heterogeneity were observed.

**Conclusion:**

This meta-analysis provides a comprehensive analysis of schistosome infection in sentinel mice across China. The estimates largely mirror the ongoing trends of transmission in terms of periods and regions. Infections were most likely to occur in April, July and October. In areas where no infected snails were reported infections in sentinel mice were still observed. Due to the presence of snails and infected wildlife, detection of schistosomes in waters using such a highly sensitive method as the deployment of sentinel mice, remains of importance in schistosomiasis monitoring. We would suggest the current criteria for transmission interruption or elimination of schistosomiasis in China be adjusted by integrating the results of sentinel mice based surveys.

## Author summary

With the continued activities of the prevention and control programme in China, the prevalence and intensity of *Schistosoma japonicum* infection have been reduced to low levels. This makes it impossible to detect any infections in humans or livestock using the traditional approach of fecal and serological testing, so as to evaluate properly the risk map of infection. However, detection of existence of schistosome cercariae in waters could be an alternative way of detecting a potential focus of transmission. We therefore performed a meta-analysis of studies performed over the last 40 years to estimate the overall infection rates of *S*. *japonicum* in sentinel mice. The estimate across China in 2004 to 2018 was 3.66%, significantly lower than in 1980 to 2003. The highest estimates were observed in Hunan, followed by Anhui and Jiangxi. Two studies conducted in Hubei in 2015 and 2016 respectively, reported infected sentinel mice where no infected snails had been reported across the province since 2014. Transmission was found be most likely in April, July and October. The estimates largely mirror the ongoing trends of *S*. *japonicum* infections in terms of periods and regions. Due to the presence of snails and other infected wildlife, detection of schistosome cercariae in waters with a highly sensitive tool remains of great importance in schistosomiasis monitoring and evaluation. To the authors’ knowledge, this is the first time that the potential threat of *S*. *japonicum* in nature, to humans and livestock has been assessed in this manner. We would suggest that the current criteria for transmission interruption or elimination of *S*. *japonicum* in China be adjusted by integrating the results of sentinel mice.

## Introduction

Schistosomiasis, caused by blood flukes of the genus *Schistosoma* (phylum *Platyhelminthes*; class *Trematoda*), is a zoonotic parasitic disease and is one of the 18 neglected tropical disease listed by the World Health Organization [1,2]. Currently, an estimated 240 million people are infected with the parasites, and about 800 million are at risk of infection in 78 tropical and subtropical countries [3,4]. The majority of human infections and morbidity are caused by three schistosome species: *Schistosoma mansoni*, *Schistosoma haematobium*, and *Schistosoma japonicum* [5]. Among three species, *Schistosoma japonicum* is considered to cause the most serious disease due to its female worms’ highest egg output and a possible longest lifespan of adult worms [6]. In China, Schistosomiasis is mainly caused by the infection of *S*. *japonicum*.

Schistosomiasis japonicum is a water-borne parasitic disease with amphibious *Oncomelania hupensis* snails serving as its intermediate host. In water infected snails release cercariae, which infect humans or animals when they have water activities nearby. After infection, schistosomula migrate to the liver where they develop and mate with an opposite-sex parasite. The paired worms then migrate into mesenteric veins where they reside and lay eggs. A fraction of the eggs are discharged out of the body with host’s stool, and then in water eggs hatch into free-swimming miracidia, which penetrate snails and then develop into cercariae [7]. Schistosomiasis japonicum was once a major public health problem in China with up to 11.6 million human cases in 1950s. After nearly 70 years of control efforts, the number of infected people has been gradually reduced to nearly 37.6 thousand in 2017 [8,9]. In 2014, the central government of China proposed a two-stage roadmap with aims to achieve transmission interruption by 2020 and then to achieve disease elimination by 2030 [10,11]. However, due to the complex lifecycle of *S*. *japonicum* and its easy colonization of new snail populations [12], the widely distributed snail habitats [9], and the existence of infected wildlife [13], schistosomiasis elimination in China remains a great challenge, and recently schistosomiasis has even been believed to be more serious than previous thought [14]. One important issue we have encountered in China is that much lower infection prevalences of *S*. *japonicum*, based on the traditional fecal and serological tests, in both humans and livestock have been frequently reported and documented [15], which may have led to no or unclear targets of further control efforts.

However, detection of existence of schistosome cercariae in waters could be an alternative way in determining potential foci of transmission, or even serve as an evaluation of current schistosomiasis situation. There are several approaches for detecting water infectivity, including use of sentinel mice or rabbits, sticking cercariae with specific membrane, detection of parasite DNA with PCR, and so on [16–18]. Among these, the sentinel mice method has been most commonly employed because of its high sensitivity and simple operability [19], and even recommended as one control measure [20]. The procedure is to put a group of 5 to 10 mice into a wire cage, which is tied with foam plastics at two ends and can float on water surface. This ensures mice to expose to water on limbs, tail and lower abdomen. The period of water exposure in practice generally lasts for a few hours per day and for two to three days. The exposed mice are then raised in the laboratory for 28 to 35 days and dissected for recovering worms or eggs [21].

This method has been employed in China over last forty years. Several research even reported an infection rate of up to 100% of *S*. *japonicum* in mice [22–30]. As infections in sentinel mice could also be an index of endemic situation in areas, we therefore performed a meta-analysis of research performed over the last 40 years to estimate the overall prevalence of *S*. *japonicum* in sentinel mice to see if it mirrors the ongoing trend of the parasite transmission in China. Subgroup analyses according to study periods, seasons and regions were also performed. To the authors’ knowledge, this is the first time to assess the potential and direct threat of *S*. *japonicum* infection in natural environments to humans and livestock. The purpose was to increase our knowledge on how to facilitate schistosomiasis control, particularly in China with low infection prevalence of the parasite in both humans and cattle [15].

## Methods

### Search strategy

A comprehensive literature search was carried out for publications published before September 1, 2018. Three Chinese and two English electronic bibliographic databases, namely China National Knowledge Infrastructure (CNKI), Wanfang, Chinese Scientific Journal Databases (VIP), PubMed and Web of Science (SCI), were searched to include all published studies that reported the infection rate of *S*. *japonicum* in sentinel mice within mainland china. We used search terms ‘Sentinel mice (or mouse)’, ‘Schistosomiasis’, and ‘China’ in the English databases and ‘shaoshu’, ‘xuexichong and/or xuexichongbing’ in the Chinese databases. No restrictions were imposed. To find additional studies, we also manually checked the relevant eligible literatures through cross-references of the identified articles in the reference lists. We did not contact authors of original studies for additional information. No attempt was made to identify unpublished studies. Full text articles were downloaded or obtained through library resources.

### Study selection

All papers were imported to the literature management software Endnote X7 to eliminate duplicated records. Two authors (CQ and HZ) independently conducted an initial screening of identified titles and abstracts and then the full-text articles were downloaded for a second screening. Studies were considered eligible only if they: (i) were carried out within mainland China; (II) were neither experimental studies nor review articles; (iii) clearly reported the time performed, as least specific to year; (iv) provided geographical location, at least specific to provinces; (v) provided numbers of dissected and infected mice, or could calculate by formula; (vi) were available in full texts. Studies were excluded if they did not fulfill any of these criteria. We deemed data regarding *S*. *japonicum* infection rates in sentinel mice from the same place at the same time as a single study, and so sometimes an article can contain several studies. If the same study data were published in both English and Chinese sources, the articles with less detailed information would be excluded from this study.

### Data extraction

The detailed characteristics of each study were extracted using a pre-designed data-collection excel form. Information was recorded as follows: surname of first author, year of publication, year of study, location of study, numbers of the infected and dissected mice.

### Data analysis

The pooled infection rate and its 95% confidence intervals (CI) of *S*. *japonicum* in sentinel mice were calculated with the Freeman-Tukey double arcsine transformation [3,31]. Besides addressing the problem of variance instability, this approach also solves the problem of confidence limits falling outside the 0 to 1 range, as transformed infection rates are weighted slightly towards 50% and thus studies with infection rates of zero or one can be included in the analysis. Forest plots were used to visualize the results of each study and the heterogeneity among studies. Between studies heterogeneity was assessed using the Cochran’s Q (reported as p values), which is quantified by I^2^ values. The I^2^ index indicate the variation between studies attributed to heterogeneity rather than chance, with values of 25, 50 and 75% corresponding to low, moderate, and high degrees of heterogeneity, respectively [32]. When there was evidence of heterogeneity (I^2^ > 50%), infection rates were combined by using a random-effects model; otherwise, rates were combined by using a fixed-effects model [33].

We also conducted subgroup analyses to investigate potential sources of heterogeneity. Such analyses were performed on the following variables: 1) by study periods, i.e. 1980 to 2003 and 2004 to 2018 according to the schistosomiasis control strategy implemented [34]; 2) by river reaches, i.e the upper, middle, and lower reaches of the Yangtze River [35]. The upper reach includes Sichuan and Yunnan provinces, the middle includes Hunan, Hubei and Jiangxi provinces, and the lower includes Anhui, Jiangsu and Zhejiang provinces; 3) by provinces; 4) by seasons a study performed. The publication bias was visually examined by funnel plots and the statistical significance was assessed by the Egger’s regression asymmetry test [36]. A two-tailed p value < 0.05 was considered statistically significant. Extracted data were entered into Microsoft Office Excel 2016 and R3.5.1 was used in all statistical analyses. This study was carried out in accordance with the Preferred Reporting Items for Systematic Reviews and Meta-analyses (PRISMA) guidelines [37], and the PRISMA checklist (S1 Checklist) was used as the basis for inclusion of relevant information.

## Results

### Literature search

**Fig 1:**
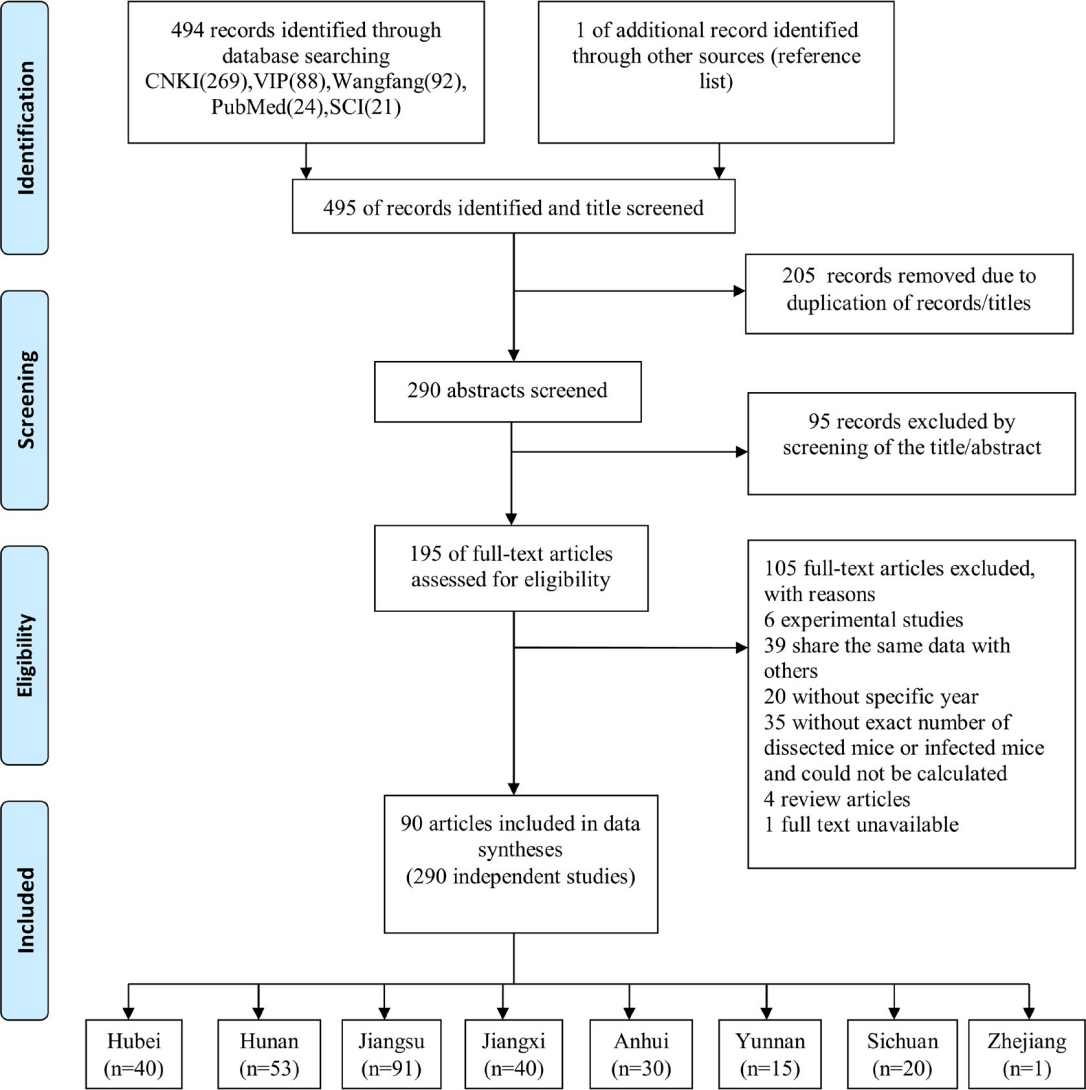
Flow chart of study selection. The diagram shows the numbers of titles and studies reviewed in preparation of this meta-analysis of *S*. *japonicum* infection rates in sentinel mice associated with infectious waters. n represents the number of studies included in data syntheses.

We identified 495 potentially relevant publications through five databases and the reference lists, of which 205 articles were excluded when taking duplication into consideration. After the initial screening of titles and abstracts, a further 95 articles were excluded. Employing the selection criteria, we obtained quantitative data for our meta-analysis after reading through full texts. The search strategy finally resulted in 90 articles (3 in English and 87 in Chinese)[22–30,38–118], reporting 290 studies. Fig 1 shows our systematic workflow for identifying, screening, and including studies in this study.

### Study characteristics

The years of the studies performed and published ranged from 1980 to 2018 and from 1985 to 2018, respectively. A total of 91 studies were reported from Jiangsu province, 53 from Hunan, 40 from Jiangxi, 40 from Hubei, 30 from Anhui, 20 from Sichuan, 15 from Yunnan, and one from Zhejiang. A total of 63998 sentinel mice were dissected and 7521 were identified with *S*. *japonicum* infection. A total of 153 studies were carried out during 1980 to 2003, and 137 during 2004 to 2018. The infection rates of *S*. *japonicum* in sentinel mice among the included studies varied between 0 and 100%. The detailed characteristics of each study are provided in Supporting Information file S1 Table, and its infection rate with 95% CI are also plot in Supporting Information file S1 Fig.

### Pooling and heterogeneity analysis

A substantial heterogeneity was observed among studies (χ^2^ = 20412.5, *p* < 0.0001; I^2^ = 98.6%, 95% CI: 98.5–98.6%). When calculated using a random-effects model, the overall infection rate was 12.31% (95% CI: 10.14–14.65%).

**Fig 2:**
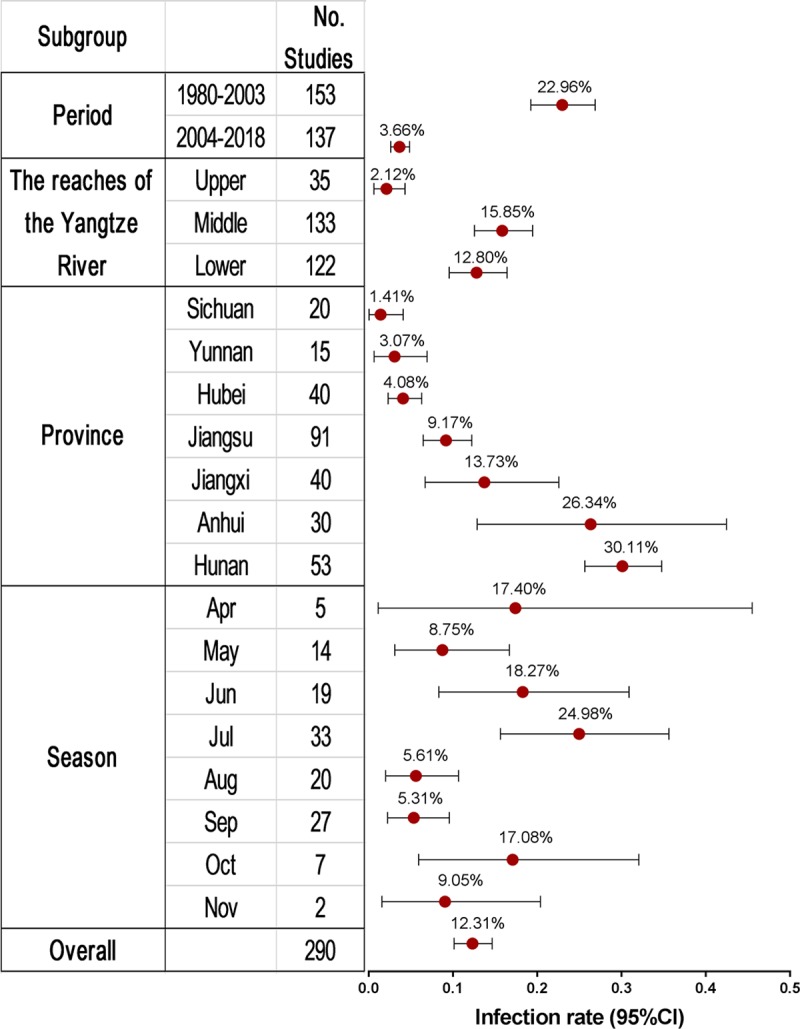
Forest plot of infection rates of *S*. *japonicum* in sentinel mice. Red circles indicate infection rate estimated by random effects meta-analysis and whisker bars indicate the 95% CI. Results are shown for all included studies (bottom line, n = 290), and for subgroups according to periods, river reaches, provinces (excluding Zhejiang due to one study only) or seasons (for studies with the time of month specified).

**Fig 3:**
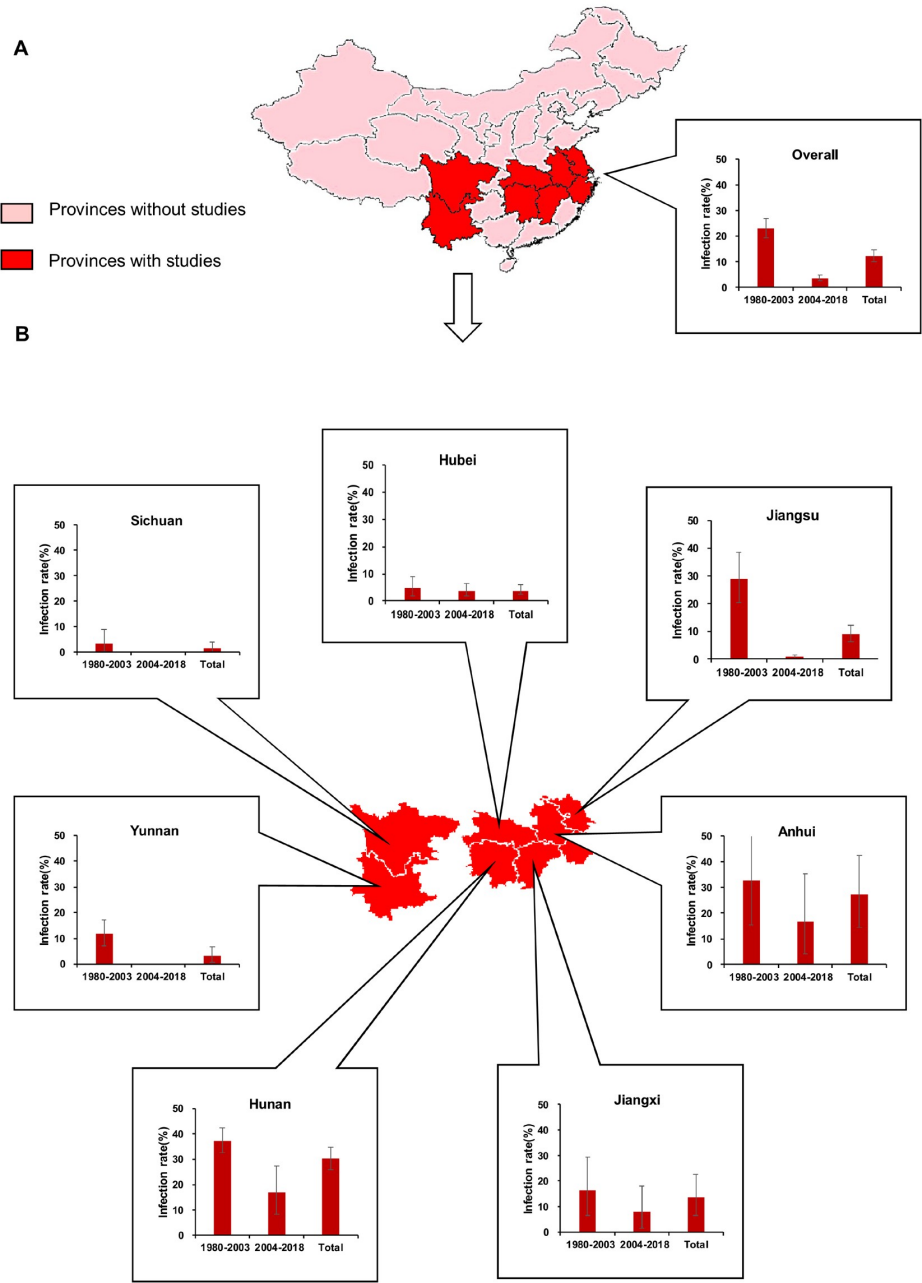
Distribution of eligible studies and pooled infection rate (95% CIs) of *S*. *japonicum* in sentinel mice. (A) across China; (B) by province and period. These data are presented numerically in Table 1 and Supporting Information file S2 Table. Map was created using R 3.5.1 and data sources was from http://www.cnblogs.com/skyme/p/5182149.html.

The estimates of infection rates for different subgroups and heterogeneities are presented in Table 1 and Fig 2. All pooled infection rates for each subgroup were calculated using a random-effects model because of the observed high heterogeneity among studies within subgroups. Based on study periods, the estimate had been significantly reduced since 2004 (1980–2003: 22.96% (95% CI: 19.25–26.89%), n = 153 vs 2004–2018: 3.66% (95% CI: 2.62–4.85%), n = 137). In terms of the River reaches, the estimate was highest in the middle reach (15.85%, 95% CI: 12.54–19.45%, n = 133), followed by that in the lower reach (12.80%, 95% CI: 9.55–16.42%, n = 122), and the lowest in the upper reach (2.12%, 95% CI: 0.63–4.32%, n = 35). At the level of provinces, the estimates ranged from 1.41% (95% CI: 0.05–4.09%, n = 20) in Sichuan to 30.11% (95% CI: 25.64–34.77%, n = 53) in Hunan. In terms of season, the estimate was highest in July (24.98%, 95% CI: 15.64–35.62%, n = 33) and lowest in September (5.36%, 95% CI: 2.25–9.57%, n = 27). The forest plots for each subgroup are provided in Supporting Information file S2–S4 Figs. In addition, we also made a further stratification of meta-analyses within provinces according to study periods. As seen in Fig 3, a rapid reduction in the pooled infection rate has been seen in all provinces since 2004 with the exception of Hubei province. Even in the latter, two studies reported infection rates of 1.75% in 2015 and 2.5% in 2016. See Supporting Information file S1 Table.

**Table 1:** Pooled infection rates of *S*. *japonicum* in sentinel mice across China and within subgroups.

Factors related to infection rate		No. of papers included	No. of studies included	No. of infected mice	No. of total mice examined	Pooled Infection Rate (95%CI)	Heterogenity			Egger's test	
							Q-χ^2^	Q-P	I^2^(%)	t	p
**Overall**		90	290	7521	63998	0.1231(0.1014–0.1465)	20412.50	<0.0001	98.6	3.97	<0.0001
**Periods**	1980–2003	49	153	6459	26667	0.2296(0.1925–0.2689)	7836.80	<0.0001	98.1	-0.17	0.8682
	2004–2018	56	137	1062	37331	0.0366(0.0262–0.0485)	3719.61	<0.0001	96.3	6.77	<0.0001
**Province**	Hubei	18	40	313	6089	0.0408(0.0230–0.0629)	520.66	<0.0001	92.5	1.45	0.1565
	Hunan	18	53	4061	14870	0.3011(0.2564–0.3477)	1642.94	<0.0001	96.8	0.48	0.6361
	Jiangsu	28	91	1325	26194	0.0917(0.0647–0.1224)	5053.29	<0.0001	98.2	5.35	<0.0001
	Jiangxi	14	40	635	4782	0.1373(0.0671–0.2256)	2348.56	<0.0001	98.3	2.71	0.0101
	Anhui	9	30	734	2772	0.2634(0.1288–0.4244)	2332.46	<0.0001	98.8	2.39	0.02381
	Yunnan	7	15	114	2237	0.0307(0.0064–0.0693)	242.40	<0.0001	94.2	0.91	0.3816
	Sichuan	6	20	339	6923	0.0141(0.0005–0.0409)	891.27	<0.0001	97.9	-0.85	0.4074
	Zhejiang	1	1	0	131						
**The reaches of the Yangtze River**	Upper	13	35	453	9160	0.0212(0.0063–0.0432)	1133.13	<0.0001	97	-0.085	0.9329
	Middle	46	133	5009	25741	0.1585(0.1254–0.1945)	7116.84	<0.0001	98.1	-1.24	0.2161
	Lower	37	122	2059	29097	0.1280(0.0955–0.1642)	8428.11	<0.0001	98.6	6.79	<0.0001
**Season**	Jan	1	1	0	8						
	Feb	1	1	0	17						
	Mar	2	2	0	169						
	Apr	3	5	171	677	0.1740(0.0113–0.4549)	247.35	<0.0001	98.4	-0.42	0.7051
	May	9	14	183	4153	0.0875(0.0309–0.1670)	696.32	<0.0001	98.1	3.33	0.0060
	Jun	15	19	466	5044	0.1827(0.0832–0.3089)	1852.19	<0.0001	99.0	3.24	0.0048
	Jul	21	33	718	6569	0.2498(0.1564–0.3562)	2650.91	<0.0001	98.8	5.11	<0.0001
	Aug	14	20	155	4252	0.0561(0.0201–0.1066)	595.90	<0.0001	96.8	3.65	0.0018
	Sep	18	27	284	6445	0.0536(0.0225–0.0957)	999.94	<0.0001	97.4	3.09	0.0049
	Oct	6	7	87	535	0.1708(0.0594–0.3205)	91.39	<0.0001	93.4	1.15	0.3027
	Nov	2	2	4	42	0.0905(0.0157–0.2038)	0.95	0.3292	0		
	Dec	1	1	0	9						

Abbreviations: CI: Confidence interval; I^2^: Inverse variance index; Q-P: Cochran’s P-value.

### Publication bias

**Fig 4:**
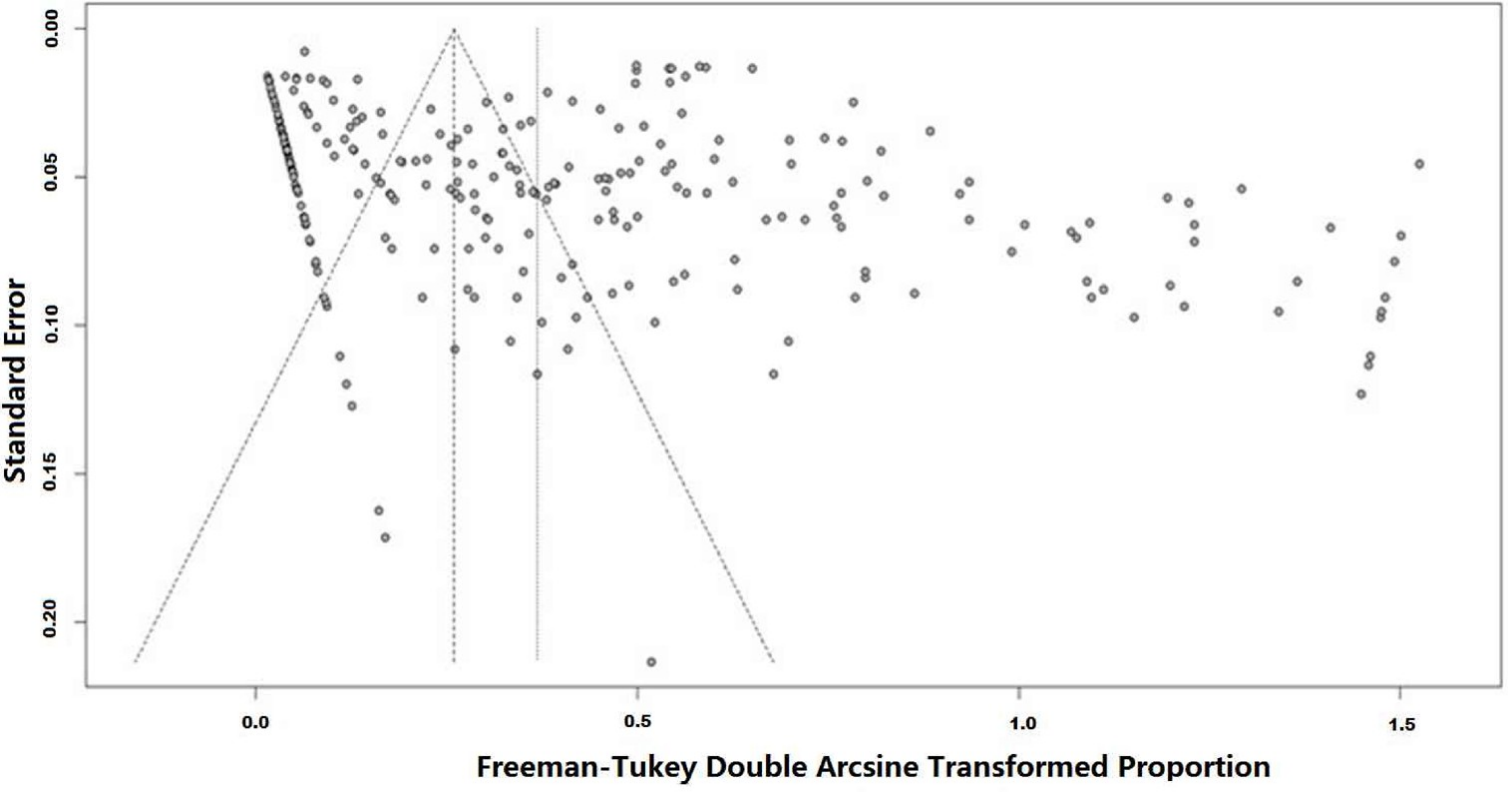
Funnel plots of the arcsine transformed infection rate of *S*. *japonicum* in sentinel mice.

A potential publication bias was indicated by Egger linear regression test (bias coefficients b = 0.16, t = 3.97, *p* < 0.0001), which is showed in Fig 4. Subgroup analyses also suggested a publication bias for studies during the period of 2004 to 2018, within each of the three provinces (i.e. Jiangsu, Jiangxi, and Anhui) and in the lower reach of the Yangtze River (see Table 1).

## Discussion

As *S*. *japonicum* prevalence in humans and livestock has been reduced to a much lower level, it seems unlikely to detect any infections with fecal and serological testing [119,120] and thus unable to accurately assessing the current status of the parasite transmission. On the contrary, on-site water surveillance for *S*. *japonicum* cercariae using sentinel mice is able to determine potential foci of transmission in nature and has been employed in China over forty years for monitoring of schistosome infection [17]. Therefore, information on the overall prevalence of *S*. *japonicum* infections in sentinel mice across China, or by time or regions, could be of implications for further control work.

This meta-analysis retrieved 90 research involving 290 field studies. The overall infection rate of *S*. *japonicum* in sentinel mice from 1980 to 2018 was 12.31% (95% CI: 10.14–14.65%), which was comparable to the infection rate of 12.24% in wild rodents in 2011 [121]. There was high heterogeneity in infection rates among studies. Such variations may partially be attributed to factors including period or season of study, and/or different river reaches or provinces. Based on study periods, the estimate significantly decreased from 22.96% in 1980 to 2003 to 3.66% in 2004 to 2018. The rapid decrease was consistent with the observation in humans [8,122] and was mainly due to the newly developed integrated control strategy [123,124], which has been, with strong political, policy and financial support, successfully implemented across China since 2004 by the national schistosomiasis control programme [125–127]. The new strategy involves a series of interventions including replacing cattle with agricultural machines, supplying clean water, providing sanitation and egg-free latrines, together with annual routine control work.

In relation to the Yangtze River, the infection rate estimate of *S*. *japonicum* in sentinel mice was significantly higher in the middle (including Hunan, Hubei and Jiangxi provinces) and lower (including Anhui, Jiangsu and Zhejiang provinces) reaches than in the upper reaches (including Sichuan and Yunnan provinces). The highest estimate was observed in Hunan (30.11%), followed by Anhui (26.34%) and then Jiangxi (13.73%). The results were in agreement with the transmission levels defined according to infections in humans and livestock [128]. The middle and lower reaches of the River belong to a subtropical climate with sufficient precipitation, where many rivers are intertwined and the two largest lakes in China (i.e. Dongting Lake in Hunan and Poyang Lake in Jiangxi) form a shallow lake group that extensively exchanges water with the Yangtze River. The water level in the lakes and in the River changes according to the monsoon season, and therefore all marshes, beaches and islands in the middle and lower reaches of the River form scenarios of so-called ‘winter-land, summer-water’. This makes such areas more suitable to intermediate host snails and most of such endemic areas are classified into the swamp and lake regions. Currently a majority of snail habitats are distributed in such regions [122]. In addition, as over forty mammal species can serve as potential infection reservoirs for *S*. *japonicum* [129], existence of any infections in wildlife, for example infected rodents in hilly areas of Anhui [13,130], would have complicated local transmission of the parasite.

All provinces, with the exception of Hubei, have seen the rapid decrease in pooled infection rates since 2004. Besides thanks to the new control strategy, the Three Gorges Dam, which is located in the upper reaches of the Yangtze River and began to function in June 2003, might play a part in reducing *S*. *japonicum* infections in the middle and lower reaches. The dam has reduced the risk of flooding and the density of living snails in the downstream regions [131]. For example, by 2015 the mean density of living snails in the Dongting Lake area has been reduced to less than 5 snails/0.11 m_2_ [132]. The changes of the water level caused by the dam, coupled by the new control strategy, might be accelerating the progress towards transmission interruption in the middle and lower reaches of the Yangtze River [133]. However, we also noted that even in 2015 and 2016 in Hubei province, infections in sentinel mice was identified [42,43], although no infected snails had been found in all surveillance sites across the province since 2014 [122]. This, together with the insignificant change in the estimate in Hubei between two periods would await further investigations.

Provinces with larger numbers of sentinel mice used may well represent the infectivity of schistosomiasis affected areas, and are believed to provide more reliable findings. Among twelve endemic provinces in southern China, we obtained a total of 289 studies from seven provinces (i.e. Jiangsu, Jiangxi, Anhui, Hubei, Hunan, Sichuan and Yunnan). In each province more than 2000 sentinel mice were dissected for *S*. *japonicum* infections. We retrieved only one study in Zhejiang province, in which no infections was reported [115]. We could not obtain any data from the other four provinces. This may be partly due to their early success in schistosomiasis control as the four provinces have each reached the level of schistosomiasis interruption with both Guangdong and Shanghai in 1985, Fujian in 1987 and Guangxi in 1989 [126].

In terms of seasons surveys conducted, the estimate was 17.4% in April, then decreased in the following months. It again rose gradually and peaked in July (24.98%). Following that it decreased and again peaked in October (17.8%). This clearly showed that infections were most likely to occur in April, July or October. Snails usually go into the soil to hibernate during cold Winter. In April, rains increases and temperature rises, rain water floods high-risk areas for the first time of a year and over-winter infected snails, when contacting the water, release a large number of cercariae. The rising temperature from April to July may facilitate the development and mature of the parasite within a snail [134], and then in July the newly developed infected snails will increase the release of more schistosome larvae, thus making the areas of affected habitats most infectious. The peak of infections in Oct. could be due to the appearance of the newly borne and infected snails developed [135].

The sentinel mice method has high sensitivity in detecting infectivity of water bodies and then determining foci of transmission. However, the main disadvantage in practice is to find a fixed point (for example a tree) on a river bank, to which a mice cage is tied. This severely restricts the range of water surface to be detected, thus reducing its operability and efficiency. This method has recently been updated by Dr. Sun at Jiangsu Institute of Parasitic Diseases, who installed a propeller motor on the mice cage and enable it to move on water under remote control. This application expands the water area, and even make it possible to detect cercariae in more complicated environments where snail surveys can not be performed. Combined with the successful detection of schistosome DNA with the PCR assay in sera of mice at three-day post-infection [136], this would provide real-time risk information on potential foci of transmission and provide early warning.

### Limitations

Though this study provided information on schistosomiasis transmission in China over the last 40 years and also directly assessed the threat of infections to humans and livestock, which would be useful in aiding future schistosomiasis control, it is not devoid of limitations. First, the estimations of infection rates using a random-effect model may not absolutely invalidate the heterogeneity between studies. Secondly, we don’t conduct the sensitivity analysis, as nearly 290 studies were included. Finally, we observed some evidence of publication bias in this work. Publication bias may exist when there is a preference to publish studies with significant findings. However, there is no certainty for a paper with high or low infection rates of *S*. *japonicum* in sentinel mice to get easily published; moreover, not all subgroup analyses showed publication bias. We thus think that publication bias is unlikely to have distorted our results.

### Conclusions

This meta-analysis provides a comprehensive analysis of *S*. *japonicum* infection in sentinel mice across China. The estimates largely mirror the ongoing trends of *S*. *japonicum* infections in terms of periods and regions. Infections were most likely to occur in April, July and October. However, in areas where no infected snails were reported infections of *S*. *japonicum* in sentinel mice were still observed. Due to the wide distribution of snails and the existence of any infected wildlife, detection of schistosome in waters using such a highly sensitive method remains of importance in the monitoring and objective evaluation of the disease. We would suggest that the current criteria for transmission interruption or elimination of *S*. *japonicum* in China [137] be adjusted by integrating the results of sentinel mice method. The recently updated method will facilitate its wide application and make the index easily obtained.

## Supporting information

S1 Checklist PRISMA checklist. (DOC)

S1 Table Characteristics of the eligible studies. (DOCX)

S2 Table Pooled infection rate estimates of *S. japonicum* in sentinel mice based on period within provinces with a random-effects analysis. (DOCX)

S1 Fig Forest plots of infection rates of *S. japonicum* in sentinel mice with a random-effects analysis. (TIFF)

S2 Fig Forest plots of infection rates of *S. japonicum* in sentinel mice by study periods with a random-effects analysis. (DOC)

S3 Fig Forest plots of infection rates of *S. japonicum* in sentinel mice by the reaches of the Yangtze River with a random-effects analysis. (DOC)

S4 Fig Forest plots of infection rates of sentinel mice by provinces with a random-effects analysis. (DOC)
