# Supplementary material for: A meta-analysis of infection rates of Schistosoma japonicum in sentinel mice associated with infectious waters in mainland China over last 40 years
Source: PLoS Negl Trop Dis. 2019 Jun 7;13(6):e0007475. doi: 10.1371/journal.pntd.0007475 (PMC6584001; doi:10.1371/journal.pntd.0007475)
Supplement: S1 Table — (DOCX) [file pntd.0007475.s002.docx]

**S1 Table.** Characteristics of the research and eligible studies. One research may include multiple studies.

| **First Author, publication year** | **The reaches of the Yangtze River** | **Province** | **Location of study** | **Year (and month) study performed** | **No. mice dissected** | **No. mice infected** | **Infection rate(%)** |
| --- | --- | --- | --- | --- | --- | --- | --- |
| Li et al, 1990 | Lower reach | Anhui | Dongdian village, Tongling country | 1980 | 22 | 9 | 40.91 |
|  |  |  |  | 1984 | 169 | 0 | 0.00 |
|  |  |  |  | 1987 | 95 | 49 | 51.58 |
|  |  |  |  | 1988 | 80 | 51 | 63.75 |
|  |  |  |  | 1989 | 94 | 32 | 34.04 |
| Jiang et al, 1991 | Lower reach | Anhui | Guifan and Sanlian village, Guichi city | Mar-May 1984 | 61 | 29 | 47.54 |
|  |  |  |  | Mar-May 1987 | 72 | 64 | 88.89 |
|  |  |  |  | Mar-May 1988 | 94 | 61 | 64.89 |
|  |  |  |  | Mar-May 1989 | 120 | 120 | 100.00 |
| Jiang et al, 1992 | Lower reach | Anhui | Jijiaba, Guichi city | 1980 | 85 | 79 | 92.94 |
|  |  |  |  | 1981 | 97 | 19 | 19.59 |
|  |  |  |  | 1982 | 149 | 2 | 1.34 |
|  |  |  |  | 1983 | 126 | 4 | 3.17 |
|  |  |  |  | 1984 | 60 | 0 | 0.00 |
|  |  |  |  | 1987 | 40 | 0 | 0.00 |
|  |  |  |  | 1988 | 30 | 0 | 0.00 |
|  |  |  |  | 1989 | 40 | 0 | 0.00 |
| Wang et al, 1995 | Lower reach | Anhui | Guanghui Village, Tongling County | July 1992 | 36 | 10 | 27.78 |
|  |  |  |  | July 1993 | 57 | 51 | 89.47 |
| Zhang et al, 1996 | Lower reach | Anhui | Dantu country | 1992 | 67 | 5 | 7.46 |
| Wang et al, 2011 | Lower reach | Anhui | Wuhu city | Jun 2009 | 57 | 41 | 71.93 |
|  |  |  |  | Jul 2009 | 60 | 26 | 43.33 |
|  |  |  |  | Aug 2009 | 60 | 26 | 43.33 |
|  |  |  |  | Sep 2009 | 60 | 39 | 65.00 |
|  |  |  |  | Oct 2009 | 60 | 11 | 18.33 |
| Yang et al, 1995 | Lower reach | Jiangsu | Xinmin village, Jianshe Township, Jiangpu Country, Nanjing city | May-Oct 1992 | 34 | 9 | 26.47 |
|  |  |  |  | May-Oct 1993 | 88 | 0 | 0.00 |
| Xu et al, 1996 | Lower reach | Jiangsu | Nanjing city | Sep 1993 | 27 | 26 | 96.30 |
|  |  |  |  | Jun 1994 | 40 | 40 | 100.00 |
|  |  |  |  | Jun 1995 | 48 | 43 | 89.58 |
| Pu et al, 1996 | Lower reach | Jiangsu | Jiangpu County, Nanjing city | 1994 | 100 | 9 | 9.00 |
| Hong et al, 1997 | Lower reach | Jiangsu | Datong village, Baguazhou township, Qixia district, Nanjing city | Jul 1993 | 97 | 18 | 18.56 |
|  |  |  |  | Jul 1994 | 92 | 2 | 2.17 |
| Qiu et al, 1998 | Lower reach | Jiangsu | Nanjing city | 22 Jul 1997 | 16 | 16 | 100.00 |
| Xu et al, 1999 | Lower reach | Jiangsu | Nanjing city | Jun 1998 | 406 | 202 | 49.75 |
|  |  |  |  | Sep 1998 | 185 | 85 | 45.95 |
| Zhou et al, 1999 | Lower reach | Jiangsu | Jiangning County, Nanjing city | 15 July 1996 | 50 | 1 | 2.00 |
| Xie et al, 2000 | Lower reach | Jiangsu | Bagua Island, Qixia district | Jul 1999 | 19 | 19 | 100.00 |
|  |  |  |  | Sep 1999 | 30 | 15 | 50.00 |
| Yang et al, 2000 | Lower reach | Jiangsu | Xiaba village, Baguazhou Island , Qixia district, Nanjing city | Jun-Jul1996 | 27 | 27 | 100.00 |
|  |  |  |  | Jun-Jul1997 | 30 | 24 | 80.00 |
| Dai et al, 2004 | Lower reach | Jiangsu | Gaozi Town, Dantu District, Zhenjiang city | May 2001 | 91 | 13 | 14.29 |
|  |  |  |  | Jun 2001 | 83 | 16 | 19.28 |
|  |  |  |  | Jul 2001 | 108 | 28 | 25.93 |
|  |  |  |  | Aug 2001 | 92 | 13 | 14.13 |
|  |  |  |  | Sep 2001 | 105 | 22 | 20.95 |
|  |  |  |  | Oct 2001 | 31 | 18 | 58.06 |
|  |  |  |  | Nov 2001 | 21 | 1 | 4.76 |
|  |  |  |  | Dec 2001 | 9 | 0 | 0.00 |
|  |  |  |  | Jan 2002 | 8 | 0 | 0.00 |
|  |  |  |  | Feb 2002 | 17 | 0 | 0.00 |
|  |  |  |  | Mar 2002 | 15 | 0 | 0.00 |
|  |  |  |  | Apr 2002 | 26 | 4 | 15.38 |
| Li et al, 2004 | Lower reach | Jiangsu | Gaochun Country | 7-8 Sep 2003 | 100 | 0 | 0.00 |
| Xie et al, 2005 | Lower reach | Jiangsu | Nanjing city | Jul 2003 | 781 | 208 | 26.63 |
|  |  |  |  | Jul 2004 | 465 | 49 | 10.54 |
| Yang et al, 2006 | Lower reach | Jiangsu | Nanjing city | Jul 2004 | 28 | 25 | 89.29 |
|  |  |  |  | Jul 2005 | 28 | 0 | 0.00 |
| Li et al, 2007 | Lower reach | Jiangsu | Gaochun County | 1999 | 39 | 0 | 0.00 |
|  |  |  |  | 2000 | 58 | 0 | 0.00 |
|  |  |  |  | 2001 | 37 | 19 | 51.35 |
|  |  |  |  | 2002 | 56 | 27 | 48.21 |
|  |  |  |  | 2005 | 90 | 10 | 11.11 |
|  |  |  |  | 2006 | 60 | 5 | 8.33 |
| Yin et al, 2008 | Lower reach | Jiangsu | Nanjing city | 2005 | 179 | 74 | 41.34 |
|  |  |  |  | 2006 | 199 | 0 | 0.00 |
|  |  |  |  | 2007 | 204 | 0 | 0.00 |
| Yang et al, 2009 | Lower reach | Jiangsu | Nanjing city | 24-25 Jul 2008 | 122 | 0 | 0.00 |
|  |  |  |  | 21-22 Aug 2008 | 193 | 0 | 0.00 |
|  |  |  |  | 17-18 Sep 2008 | 187 | 0 | 0.00 |
| Yu et al, 2010 | Lower reach | Jiangsu | yangzhong city | May-Sep 2009 | 300 | 1 | 0.33 |
| Xie et al, 2010 | Lower reach | Jiangsu | Nanjing city | 1999 | 146 | 78 | 53.42 |
|  |  |  |  | 2000 | 142 | 14 | 9.86 |
|  |  |  |  | 2001 | 25 | 3 | 12.00 |
|  |  |  |  | 2002 | 32 | 2 | 6.25 |
|  |  |  |  | 2006 | 29 | 0 | 0.00 |
|  |  |  |  | 2007 | 30 | 0 | 0.00 |
| Sun et al, 2011 | Lower reach | Jiangsu | Nanjing, Zhenjiang, Yangzhou, Changzhou and Taizhou city | 12-13 May 2009 | 886 | 2 | 0.23 |
|  |  |  |  | 10-11 June 2009 | 866 | 2 | 0.23 |
|  |  |  |  | 10-11 July 2009 | 866 | 0 | 0.00 |
|  |  |  |  | 10-11 Aug 2009 | 886 | 4 | 0.45 |
|  |  |  |  | 6-7 Sept 2009 | 866 | 15 | 1.73 |
|  |  |  |  | 30-31 May 2010 | 829 | 6 | 0.72 |
|  |  |  |  | 18-19 June 2010 | 737 | 6 | 0.81 |
|  |  |  |  | 14-15 July 2010 | 795 | 0 | 0.00 |
|  |  |  |  | 12-13 Aug 2010 | 652 | 0 | 0.00 |
|  |  |  |  | 9-10 Sept 2010 | 830 | 0 | 0.00 |
| Xie et al, 2011 | Lower reach | Jiangsu | Nanjing city | Jul 2001 | 115 | 18 | 15.65 |
|  |  |  |  | Jul 2002 | 179 | 58 | 32.40 |
|  |  |  |  | Jul 2006 | 146 | 0 | 0.00 |
|  |  |  |  | Jul 2007 | 149 | 0 | 0.00 |
| Jiang et al, 2012 | Lower reach | Jiangsu | Gaoqiao Town, Dantu District, Zhenjiang city | Jul-Sep 2005 | 60 | 12 | 20.00 |
| Yang et al, 2013 | Lower reach | Jiangsu | - | May-Sep 2011 | 4503 | 18 | 0.40 |
| Ren et al, 2013 | Lower reach | Jiangsu | Xinmin village, Gaoyou city | May-Sep 2012 | 200 | 0 | 0.00 |
| Sun et al, 2015 | Lower reach | Jiangsu | Nanjing, Zhenjiang, Yangzhou, Changzhou and Taizhou city | May 2014 | 980 | 0 | 0.00 |
|  |  |  |  | Jun 2014 | 995 | 0 | 0.00 |
|  |  |  |  | Jul 2014 | 981 | 0 | 0.00 |
|  |  |  |  | Aug 2014 | 984 | 0 | 0.00 |
|  |  |  |  | Sep 2014 | 993 | 1 | 0.10 |
| Wan et al, 2015 | Lower reach | Jiangsu | Nanjing city | Sep 2009 | 37 | 4 | 10.81 |
| Zuo et al, 2016 | Lower reach | Jiangsu | Yangzhou city | May 2015 | 296 | 0 | 0.00 |
|  |  |  |  | Jun 2015 | 299 | 0 | 0.00 |
|  |  |  |  | Jul 2015 | 296 | 0 | 0.00 |
|  |  |  |  | Aug 2015 | 300 | 0 | 0.00 |
|  |  |  |  | Sep 2015 | 299 | 0 | 0.00 |
| Shen et al, 2017 | Lower reach | Jiangsu | Wudun Village, Jiangxin Township, Dantu District, Zhenjiang city | May-Sep 2004 | 30 | 3 | 10.00 |
|  |  |  |  | May-Sep 2005 | 30 | 2 | 6.67 |
|  |  |  |  | May-Sep 2006 | 30 | 1 | 3.33 |
|  |  |  |  | May-Sep 2007 | 30 | 5 | 16.67 |
|  |  |  |  | May-Sep 2008 | 30 | 2 | 6.67 |
|  |  |  |  | May-Sep 2012 | 84 | 0 | 0.00 |
|  |  |  |  | May-Sep 2013 | 100 | 0 | 0.00 |
|  |  |  |  | May-Sep 2015 | 100 | 0 | 0.00 |
|  |  |  |  | May-Sep 2016 | 40 | 0 | 0.00 |
| Tao et al, 1989 | Lower reach | Zhejiang | Zhuangbu Village, Xingkou Township, Kaihua County | 1987 | 131 | 0 | 0.00 |
| Zhan et al, 1995 | Middle reach | Hubei | Wuhan city | Jul-Aug 1990 | 70 | 0 | 0.00 |
|  |  |  |  | Jul-Aug 1991 | 94 | 6 | 6.38 |
|  |  |  |  | Jul-Aug 1992 | 81 | 5 | 6.17 |
| Li et al, 1996 | Middle reach | Hubei | Wuhan city | Jul-Aug 1993 | 338 | 17 | 5.03 |
| Liu et al, 1996 | Middle reach | Hubei | Longwan town, qianjiang city | 1994 | 105 | 23 | 21.90 |
|  |  |  |  | 1995 | 62 | 0 | 0.00 |
| Shen et al, 1997 | Middle reach | Hubei | Xiong kou town, qianjiang city | 1992 | 80 | 10 | 12.50 |
|  |  |  |  | 1993 | 80 | 6 | 7.50 |
|  |  |  |  | 1994 | 80 | 2 | 2.50 |
|  |  |  |  | 1995 | 80 | 1 | 1.25 |
| Cao et al, 2007 | Middle reach | Hubei | Jingzhou city | May 2005 | 77 | 5 | 6.49 |
|  |  |  |  | Jun 2005 | 61 | 5 | 8.20 |
|  |  |  |  | Jul 2005 | 110 | 12 | 10.91 |
|  |  |  |  | Aug 2005 | 88 | 12 | 13.64 |
|  |  |  |  | Sep 2005 | 56 | 12 | 21.43 |
|  |  |  |  | Oct 2005 | 18 | 2 | 11.11 |
|  |  |  |  | Nov 2005 | 21 | 3 | 14.29 |
| Wang et al, 2010 | Middle reach | Hubei | Hanchuan city | 2005 | 45 | 2 | 4.44 |
|  |  |  |  | 2006 | 45 | 3 | 6.67 |
| He et al, 2011 | Middle reach | Hubei | Gong’an and Jianli counties, Jingzhou city | Jun-Jul 2010 | 120 | 0 | 0.00 |
| Tu et al, 2012 | Middle reach | Hubei | - | July 2010 | 410 | 36 | 8.78 |
|  |  |  | - | Sept 2010 | 200 | 11 | 5.50 |
| Wang et al, 2012 | Middle reach | Hubei | Wuhan city | Jun-Jul 2011 | 540 | 75 | 13.89 |
| Li et al, 2014 | Middle reach | Hubei | - | 2012 | 397 | 0 | 0.00 |
|  |  |  | - | 2013 | 229 | 3 | 1.31 |
| Xiang et al, 2016 | Middle reach | Hubei | Hanchuan city | Jun 2014 | 254 | 0 | 0.00 |
|  |  |  |  | Sep 2014 | 256 | 4 | 1.56 |
| Wang et al, 2016 | Middle reach | Hubei | Jiangxia District, Wuhan city | Jul 2014 | 40 | 0 | 0.00 |
| Li et al, 2017 | Middle reach | Hubei | - | May-June 2016 | 200 | 5 | 2.50 |
|  |  |  | - | Aug 2016 | 160 | 0 | 0.00 |
| Li et al, 2017 | Middle reach | Hubei | - | June-Aug 2015 | 285 | 5 | 1.75 |
|  |  |  | - | Oct 2015 | 103 | 0 | 0.00 |
| Li et al, 2017 | Middle reach | Hubei | Wuhan city | 2014 | 300 | 0 | 0.00 |
|  |  |  |  | 2015 | 163 | 0 | 0.00 |
|  |  |  |  | 2016 | 260 | 0 | 0.00 |
| Wang et al, 2018 | Middle reach | Hubei | Wuhan city | Apr-Oct 2017 | 219 | 0 | 0.00 |
| Liu et al, 2014 | Middle reach | Hubei | Gong'an country | 2009 | 62 | 14 | 22.58 |
|  |  |  |  | 2011 | 120 | 9 | 7.50 |
|  |  |  |  | 2011 | 120 | 2 | 1.67 |
|  |  | Hunan | Yuanjiang city | 2009 | 129 | 41 | 31.78 |
|  |  |  |  | 2010 | 124 | 8 | 6.45 |
|  |  |  |  | 2011 | 84 | 0 | 0.00 |
| Xu et al, 1988 | Middle reach | Hunan | Hanshou country | 1984 | 62 | 25 | 40.32 |
| Tang et al, 1991 | Middle reach | Hunan | Changde city | Apr-May 1985 | 233 | 55 | 23.61 |
| Pang et al, 1992 | Middle reach | Hunan | Anxiang County | 1985 | 85 | 5 | 5.88 |
| Zhang et al, 1993 | Middle reach | Hunan | Xingfu Township, Huarong County | Jul 1985 | 26 | 26 | 100.00 |
|  |  |  |  | Jul 1986 | 20 | 20 | 100.00 |
|  |  |  |  | Jul 1987 | 32 | 26 | 81.25 |
| Nie et al, 1994 | Middle reach | Hunan | Huarong County, Yueyang city | 10-12 Jul 1991 | 55 | 54 | 98.18 |
| Liao et al, 1996 | Middle reach | Hunan | Huarong County, Yueyang city | 1984 | 163 | 10 | 6.13 |
|  |  |  |  | 1985 | 90 | 4 | 4.44 |
|  |  |  |  | 1986 | 45 | 4 | 8.89 |
|  |  |  |  | 1987 | 22 | 2 | 9.09 |
|  |  |  |  | 1988 | 34 | 27 | 79.41 |
|  |  |  |  | 1989 | 44 | 31 | 70.45 |
|  |  |  |  | 1991 | 41 | 14 | 34.15 |
|  |  |  |  | 1992 | 32 | 11 | 34.38 |
|  |  |  |  | 1993 | 35 | 5 | 14.29 |
| Wu et al, 1996 | Middle reach | Hunan | Junshan District, Yueyang city | 1993 | 35 | 18 | 51.43 |
|  |  |  |  | 1995 | 50 | 4 | 8.00 |
| Zhang et al, 1998 | Middle reach | Hunan | Yueyang County | 1992 | 70 | 33 | 47.14 |
|  |  |  |  | 1993 | 33 | 29 | 87.88 |
|  |  |  |  | 1994 | 33 | 7 | 21.21 |
|  |  |  |  | 1995 | 31 | 6 | 19.35 |
| Chen et al, 1998 | Middle reach | Hunan | Dongting Lake District | Apr-May 1987 | 1249 | 286 | 2.50 |
|  |  |  |  | Apr-May 1988 | 1402 | 514 | 2.50 |
|  |  |  |  | Apr-May 1989 | 1426 | 379 | 2.50 |
|  |  |  |  | Apr-May 1990 | 1468 | 454 | 2.50 |
|  |  |  |  | Apr-May 1991 | 1537 | 463 | 2.50 |
|  |  |  |  | Apr-May 1992 | 969 | 276 | 2.50 |
|  |  |  |  | Apr-May 1993 | 1637 | 374 | 2.50 |
|  |  |  |  | Apr-May 1994 | 1396 | 375 | 2.50 |
| Xie et al, 2001 | Middle reach | Hunan | Yueyang city | Aug-Sep 1998 | 310 | 87 | 28.06 |
| Xie et al, 2002 | Middle reach | Hunan | Yueyang city | May 1999 | 81 | 39 | 48.15 |
|  |  |  |  | Jun 1999 | 50 | 39 | 78.00 |
|  |  |  |  | Jul 1999 | 78 | 42 | 53.85 |
|  |  |  |  | Aug 1999 | 81 | 9 | 11.11 |
| Yang et al, 2009 | Middle reach | Hunan | Junshan District, Yueyang city | Jun 2008 | 26 | 22 | 84.00 |
|  |  |  |  | Jul 2008 | 34 | 33 | 97.06 |
|  |  |  |  | Aug 2008 | 18 | 7 | 38.89 |
| Liu et al, 2010 | Middle reach | Hunan | Fengjiawan Village, Yuanjiang city | Aug 2008 | 20 | 0 | 0.00 |
|  |  |  |  | Aug 2009 | 20 | 0 | 0.00 |
| Liu et al, 2017 | Middle reach | Hunan | Junshan District, Yueyang city | 2009 | 120 | 50 | 41.67 |
|  |  |  |  | 2010 | 120 | 32 | 26.67 |
|  |  |  |  | 2011 | 82 | 10 | 12.20 |
|  |  |  |  | 2012 | 79 | 2 | 2.53 |
|  |  |  |  | 2015 | 90 | 0 | 0.00 |
| Hu et al, 1985 | Middle reach | Jiangxi | Sanli Township, Jinxian Country | May 1981 | 174 | 84 | 48.28 |
|  |  |  |  | May 1982 | 120 | 0 | 0.00 |
|  |  |  |  | Mar 1982 | 154 | 0 | 0.00 |
|  |  |  |  | May 1983 | 123 | 4 | 3.25 |
|  |  |  |  | Sep 1983 | 118 | 0 | 0.00 |
|  |  |  |  | May 1984 | 221 | 0 | 0.00 |
|  |  |  |  | Sep 1984 | 102 | 0 | 0.00 |
| Zhang et al, 1988 | Middle reach | Jiangxi | Tuniu village, Xingzi Country | 1982 | 77 | 67 | 87.01 |
|  |  |  |  | 1983 | 88 | 24 | 27.27 |
|  |  |  |  | 1985 | 75 | 10 | 13.33 |
|  |  |  |  | 1986 | 57 | 0 | 0.00 |
| Xu et al, 1989 | Middle reach | Jiangxi | Ertang Township, Jinxian Country | 1985 | 5 | 1 | 20.00 |
|  |  |  |  | 1986 | 48 | 0 | 0.00 |
|  |  |  |  | 1987 | 49 | 0 | 0.00 |
|  |  |  |  | 1988 | 37 | 0 | 0.00 |
| Zhu et al, 1991 | Middle reach | Jiangxi | Yangzi Township, Pengze Country | Apr 1987 | 209 | 125 | 59.81 |
|  |  |  |  | Apr 1988 | 126 | 29 | 23.02 |
|  |  |  |  | Apr 1989 | 135 | 1 | 0.74 |
| Jiang et al, 1996 | Middle reach | Jiangxi | Xingzi country | spring 1987 | 51 | 51 | 2.50 |
|  |  |  |  | spring 1988 | 53 | 41 | 2.50 |
|  |  |  |  | spring 1989 | 39 | 6 | 2.50 |
|  |  |  |  | spring 1992 | 30 | 30 | 2.50 |
|  |  |  |  | spring 1993 | 25 | 6 | 2.50 |
|  |  |  |  | spring 1994 | 52 | 6 | 2.50 |
| Wang et al, 1997 | Middle reach | Jiangxi | Jiujiang Country | Aug 1992 | 81 | 25 | 30.86 |
|  |  |  |  | Aug 1993 | 100 | 0 | 0.00 |
| Yu et al, 2004 | Middle reach | Jiangxi | Duchang Country | May 2001 | 130 | 6 | 4.62 |
|  |  |  |  | May 2002 | 126 | 5 | 3.97 |
| Wang et al, 2007 | Middle reach | Jiangxi | Xingzi country | May 2005 | 19 | 19 | 100.00 |
| Chen et al, 2009 | Middle reach | Jiangxi | Sanli Township, Jinxian Country | Aug 2005 | 58 | 46 | 79.31 |
|  |  |  |  | Aug 2006 | 45 | 1 | 2.22 |
|  |  |  |  | Aug 2007 | 61 | 0 | 0.00 |
|  |  |  |  | Aug 2008 | 110 | 0 | 0.00 |
| Zeng et al, 2012 | Middle reach | Jiangxi | Sanli Township, Jinxian Country | Aug 2010 | 86 | 0 | 0.00 |
|  |  |  |  | Aug 2011 | 81 | 0 | 0.00 |
| Huang et al, 2012 | Middle reach | Jiangxi | Nanchang Country | Jun-Jul 2011 | 577 | 1 | 0.17 |
| Xu et al, 1996 | Upper reach | Sichuan | Chuanxing district, Xichang city | 1987 | 743 | 169 | 22.75 |
|  |  |  |  | 1989 | 342 | 65 | 19.01 |
|  |  |  |  | 1992 | 421 | 68 | 16.15 |
|  |  |  |  | 1994 | 220 | 22 | 10.00 |
|  |  |  |  | 1995 | 121 | 0 | 0.00 |
| Zhao et al, 1998 | Upper reach | Sichuan | Qionghai Lake, Xichang city | Jul 1994 | 204 | 0 | 0.00 |
|  |  |  |  | Jun 1995 | 181 | 0 | 0.00 |
|  |  |  |  | Jul 1996 | 167 | 1 | 0.60 |
| Yang et al, 2000 | Upper reach | Sichuan | Chengdu, Meishan, Leshan city | Apr-Sep 1996 | 320 | 8 | 2.50 |
|  |  |  |  | Apr-Sep 1997 | 340 | 5 | 1.47 |
|  |  |  |  | Apr-Sep 1998 | 345 | 0 | 0.00 |
|  |  |  |  | Apr-Sep 1999 | 348 | 0 | 0.00 |
| Wu et al, 2014 | Upper reach | Sichuan | Ya’an city | Jun 2013 | 100 | 0 | 0.00 |
| Wang et al, 2014 | Upper reach | Sichuan | Lushan County | Sep 2013 | 107 | 0 | 0.00 |
| Chen et al, 2017 | Upper reach | Sichuan | Chengdu, Meishan, Deyang, Mianyang, Ya'an, Liangshan and Leshan city | 2010 | 315 | 1 | 0.32 |
|  |  |  |  | 2012 | 562 | 0 | 0.00 |
|  |  |  |  | 2013 | 502 | 0 | 0.00 |
|  |  |  |  | 2014 | 432 | 0 | 0.00 |
|  |  |  |  | 2015 | 514 | 0 | 0.00 |
|  |  |  |  | 2016 | 639 | 0 | 0.00 |
| Wu et al, 1991 | Upper reach | Yunnan | Zhonghe village, Weishan country | Oct 1988 | 143 | 14 | 9.79 |
|  |  |  |  | Oct 1989 | 81 | 23 | 28.40 |
| Yang et al, 1992 | Upper reach | Yunnan | Zhonghe village, Weishan country | Apr 1988 | 181 | 12 | 6.63 |
|  |  |  |  | Jun 1988 | 260 | 32 | 12.31 |
|  |  |  |  | Aug 1988 | 116 | 12 | 10.34 |
|  |  |  |  | Oct 1988 | 99 | 19 | 19.19 |
| Liu et al, 1997 | Upper reach | Yunnan | Zhonghe village, Weishan country | Jun-Aug 1995 | 75 | 2 | 2.67 |
| Shen et al, 2015 | Upper reach | Yunnan | - | Jun-Jul, 2014 | 134 | 0 | 0.00 |
|  |  |  | - | Sep-Sep, 2014 | 125 | 0 | 0.00 |
| Shen et al, 2017 | Upper reach | Yunnan | Dali city, Eryuan and Weishan country | Jul-Aug,2015 | 140 | 0 | 0.00 |
|  |  |  |  | Oct-Nov,2015 | 108 | 0 | 0.00 |
| Jia et al, 2010 | Lower reach | Anhui | - | Jul-Aug 2008 | 152 | 2 | 1.32 |
|  | Middle reach | Hubei | - | Jul-Aug 2008 | 60 | 23 | 38.33 |
|  | Middle reach | Hunan | - | Jul-Aug 2008 | 65 | 13 | 20.00 |
|  | Middle reach | Jiangxi | - | Jul-Aug 2008 | 165 | 42 | 25.45 |
| Zheng et al, 2012 | Lower reach | Anhui | - | Jun 2010 | 99 | 2 | 2.02 |
|  |  |  | - | Sep 2010 | 182 | 2 | 1.10 |
|  | Middle reach | Hunan | - | Jun 2010 | 218 | 16 | 7.34 |
|  |  |  | - | Sep 2010 | 221 | 46 | 20.81 |
|  | Middle reach | Jiangxi | - | Sep 2010 | 172 | 0 | 2.50 |
|  | Upper reach | Yunnan | - | Jun 2010 | 264 | 0 | 0.00 |
|  |  |  | - | Sep 2010 | 220 | 0 | 0.00 |
| Zheng et al, 2013 | Lower reach | Anhui | - | Jun-Jul 2012 | 223 | 0 | 0.00 |
|  |  |  | - | Sep 2012 | 225 | 0 | 0.00 |
|  | Lower reach | Jiangsu | - | Jun-Jul 2012 | 144 | 0 | 0.00 |
|  |  |  | - | Sep 2012 | 126 | 0 | 0.00 |
|  | Middle reach | Hunan | - | Jun-Jul 2012 | 236 | 27 | 11.44 |
|  |  |  | - | Sep 2012 | 229 | 1 | 0.44 |
|  | Middle reach | Jiangxi | - | Jun-Jul 2012 | 431 | 4 | 0.93 |
|  |  |  | - | Sep 2012 | 372 | 1 | 0.27 |
|  | Upper reach | Yunnan | - | Jun-Jul 2012 | 151 | 0 | 0.00 |
|  |  |  | - | Sep 2012 | 140 | 0 | 0.00 |

Note: ‘-’ indicates the detailed location of study was not reported in the paper.
