# Supplementary material for: A meta-analysis of infection rates of Schistosoma japonicum in sentinel mice associated with infectious waters in mainland China over last 40 years
Source: PLoS Negl Trop Dis. 2019 Jun 7;13(6):e0007475. doi: 10.1371/journal.pntd.0007475 (PMC6584001; doi:10.1371/journal.pntd.0007475)
Supplement: S2 Table — (DOCX) [file pntd.0007475.s003.docx]

**S2 Table. Pooled infection rate estimates of sentinel mice in china based on different province and period.** These data are presented graphically in Figure 3 of the paper.

| **Province** | **Period** | **No. of papers included** | **No. of studies included** | **No.of positive sentinel mice** | **No. of total sentinel mice** | **Pooled**  **infection**  **rate(95%CI)** |
| --- | --- | --- | --- | --- | --- | --- |
| **Hubei** | 1980-2003 | 4 | 10 | 70 | 1070 | 0.0497(0.0199-0.0902) |
|  | 2004-2018 | 14 | 30 | 243 | 5019 | 0.0384(0.0182-0.0645) |
|  | Total | 18 | 40 | 313 | 6089 | 0.0408(0.0230-0.0629) |
| **Hunan** | 1980-2003 | 11 | 35 | 3753 | 12955 | 0.3734(0.3259-0.4221) |
|  | 2004-2018 | 7 | 18 | 308 | 1915 | 0.1678(0.0830-0.2733) |
|  | Total | 18 | 53 | 4061 | 14870 | 0.3011(0.2564-0.3477) |
| **Jiangsu** | 1980-2003 | 15 | 40 | 1078 | 3605 | 0.2906(0.2042-0.3848) |
|  | 2004-2018 | 17 | 51 | 247 | 22589 | 0.0091(0.0035-0.0165) |
|  | Total | 28 | 91 | 1325 | 26194 | 0.0917(0.0647-0.1224) |
| **Jiangxi** | 1980-2003 | 7 | 28 | 521 | 2605 | 0.1647(0.0665-0.2926) |
|  | 2004-2018 | 7 | 12 | 114 | 2177 | 0.0797(0.0157-0.1811) |
|  | Total | 14 | 40 | 635 | 4782 | 0.1373(0.0671-0.2256) |
| **Anhui** | 1980-2003 | 5 | 20 | 585 | 1594 | 0.3162(0.1308-0.5372) |
|  | 2004-2018 | 4 | 10 | 149 | 1178 | 0.1671(0.0406-0.3519) |
|  | Total | 9 | 30 | 734 | 2772 | 0.2634(0.1288-0.4244) |
| **Yunnan** | 1980-2003 | 3 | 7 | 114 | 955 | 0.1166(0.0707-0.1718) |
|  | 2004-2018 | 4 | 8 | 0 | 1282 | 0.0000(0.0000-0.0017) |
|  | Total | 7 | 15 | 114 | 2237 | 0.0307(0.0064-0.0693) |
| **Sichuan** | 1980-2003 | 3 | 12 | 338 | 3752 | 0.0331(0.0034-0.0881) |
|  | 2004-2018 | 3 | 8 | 1 | 3171 | 0.0000(0.0000-0.0006) |
|  | Total | 6 | 20 | 339 | 6923 | 0.0141(0.0005-0.0409) |
