# Supplementary figures and images for: A meta-analysis of infection rates of Schistosoma japonicum in sentinel mice associated with infectious waters in mainland China over last 40 years
Source: PLoS Negl Trop Dis. 2019 Jun 7;13(6):e0007475. doi: 10.1371/journal.pntd.0007475 (PMC6584001; doi:10.1371/journal.pntd.0007475)

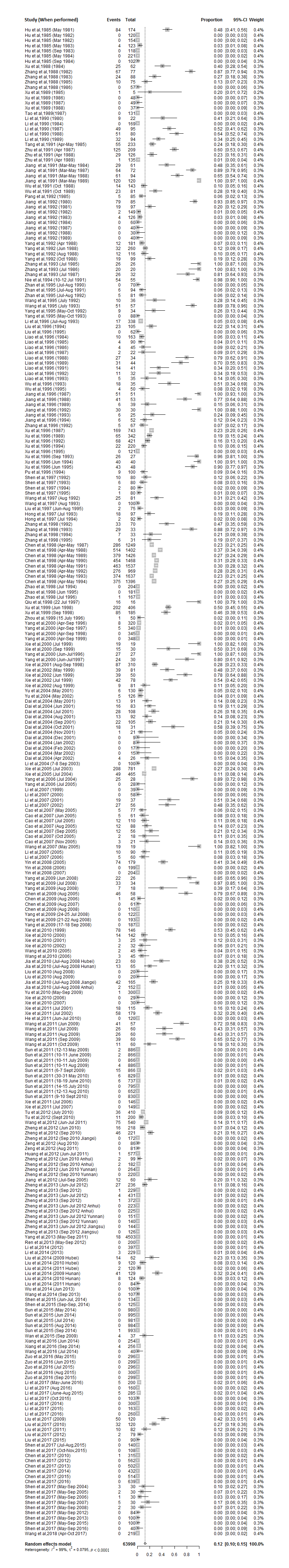

Supplement: S1 Fig — (TIFF) [file pntd.0007475.s004.tiff]
