# Supplementary material for: A meta-analysis of infection rates of Schistosoma japonicum in sentinel mice associated with infectious waters in mainland China over last 40 years
Source: PLoS Negl Trop Dis. 2019 Jun 7;13(6):e0007475. doi: 10.1371/journal.pntd.0007475 (PMC6584001; doi:10.1371/journal.pntd.0007475)

**Fig S2.1** Forest plot of infection rate of *S. japonicum* in sentinel mice in the period 1980-2003 with a random-effects analysis

**Fig S2.2** Forest plot of infection rate of *S. japonicum* in sentinel mice in the period 2004-2018 with a random-effects analysis.


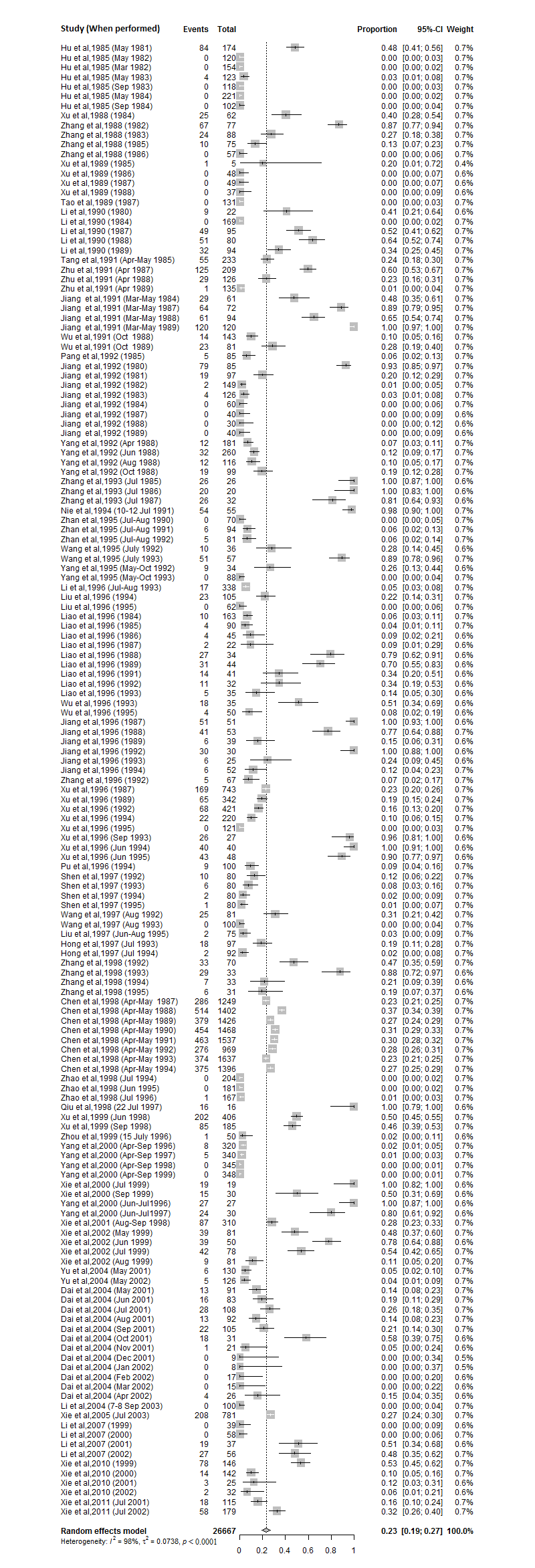


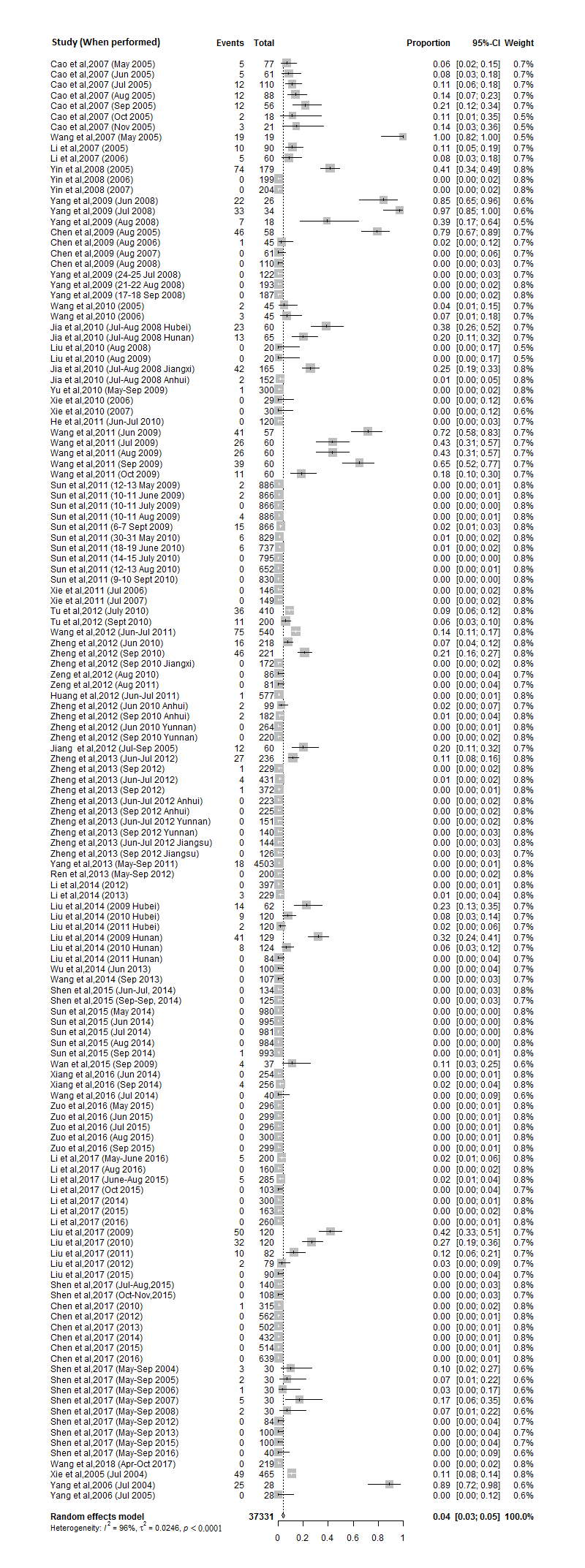

Supplement: S2 Fig — (DOC) [file pntd.0007475.s005.doc]
