# Supplementary material for: A meta-analysis of infection rates of Schistosoma japonicum in sentinel mice associated with infectious waters in mainland China over last 40 years
Source: PLoS Negl Trop Dis. 2019 Jun 7;13(6):e0007475. doi: 10.1371/journal.pntd.0007475 (PMC6584001; doi:10.1371/journal.pntd.0007475)

**Fig S3.1** Forest plot of infection rate of *S. japonicum* in sentinel mice in the upper reaches of Yangtze with a random-effects analysis.

**Fig S3.2** Forest plot of infection rate of *S. japonicum* in sentinel mice in the middle reaches of Yangtze with a random-effects analysis.

**Fig S3.3** Forest plot of infection rate of *S. japonicum* in sentinel mice in the lower reaches of Yangtze with a random-effects analysis.


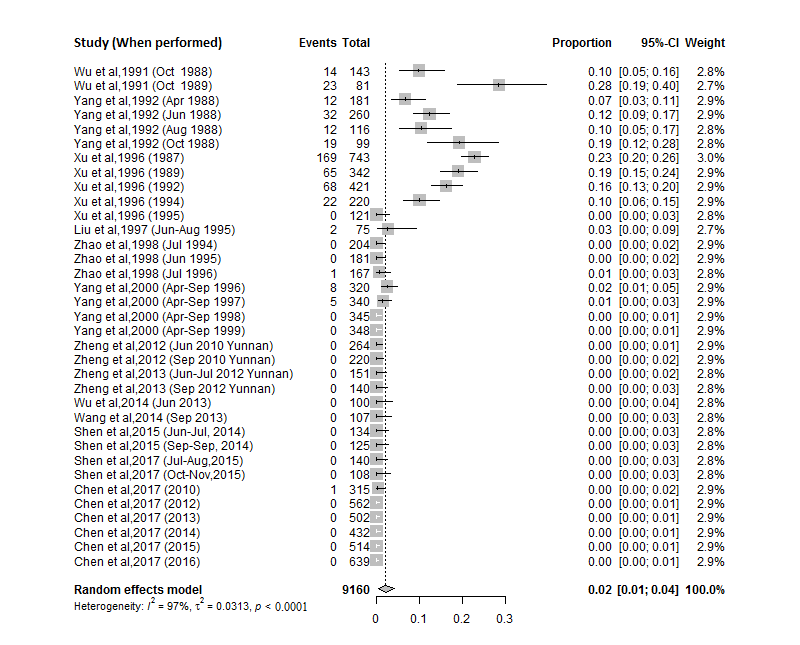


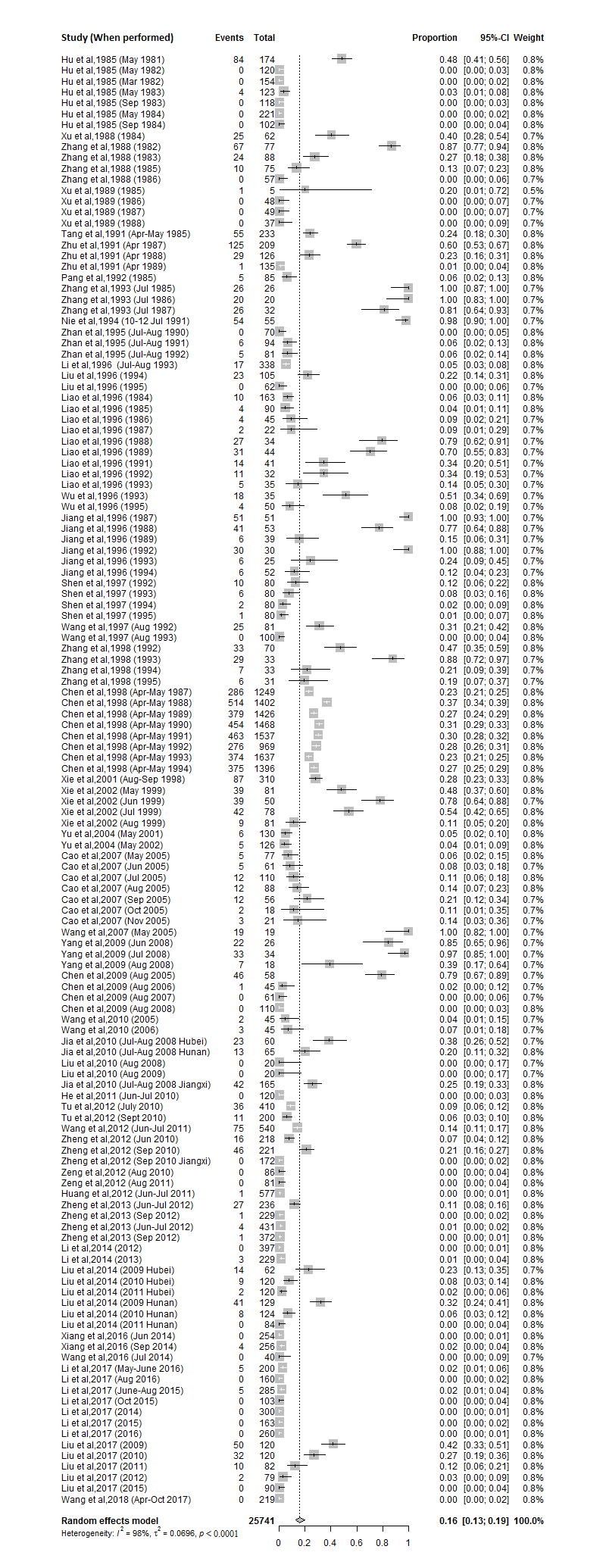


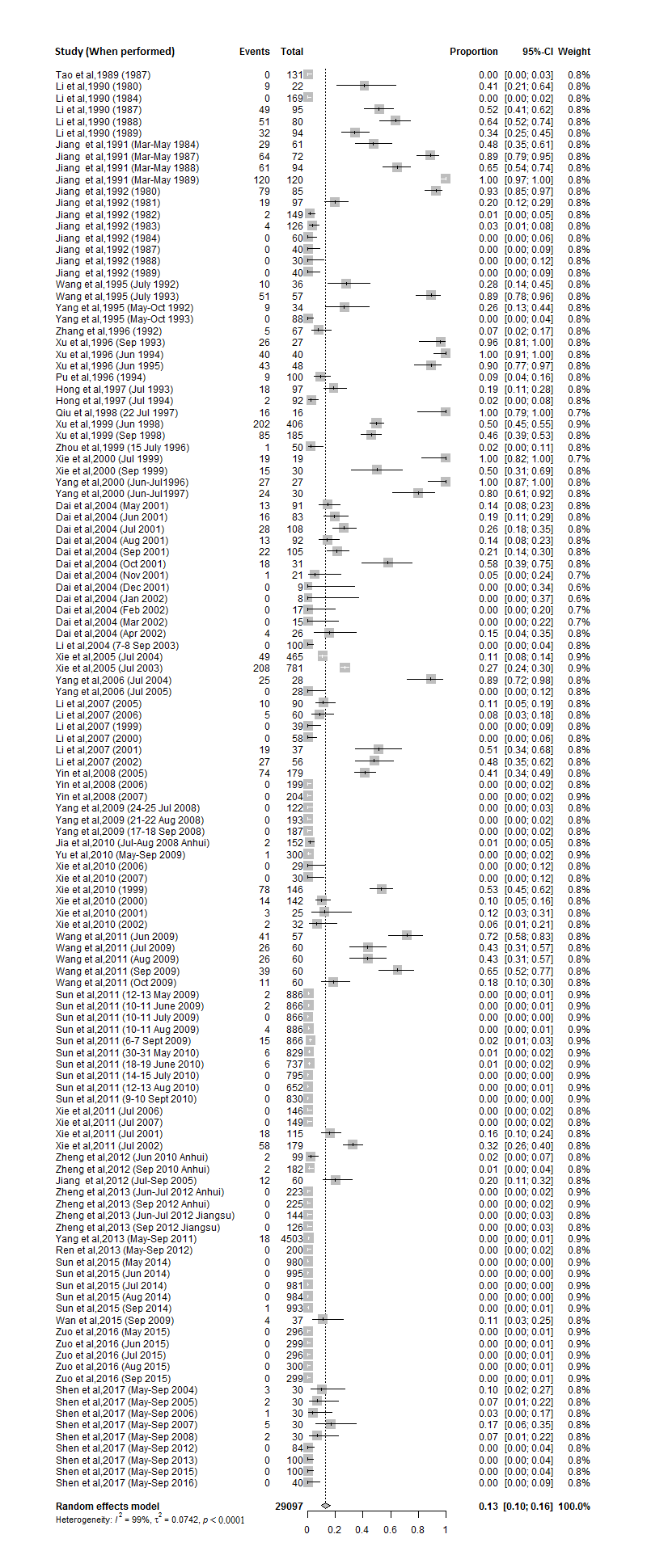

Supplement: S3 Fig — (DOC) [file pntd.0007475.s006.doc]
