# Supplementary material for: A meta-analysis of infection rates of Schistosoma japonicum in sentinel mice associated with infectious waters in mainland China over last 40 years
Source: PLoS Negl Trop Dis. 2019 Jun 7;13(6):e0007475. doi: 10.1371/journal.pntd.0007475 (PMC6584001; doi:10.1371/journal.pntd.0007475)

**Fig S4.1** Forest plot of infection rate of *S. japonicum* in sentinel mice in Hunan province with a random-effects analysis.

**Fig S4.2** Forest plot of infection rate of *S. japonicum* in sentinel mice in Anhui province with a random-effects analysis.

**Fig S4.3** Forest plot of infection rate of *S. japonicum* in sentinel mice in Jiangxi province with a random-effects analysis.

**Fig S4.3** Forest plot of infection rate of *S. japonicum* in sentinel mice in Jiangsu province with a random-effects analysis.

**Fig S4.5** Forest plot of infection rate of *S. japonicum* in sentinel mice in Hubei province with a random-effects analysis.

**Fig S4.6** Forest plot of infection rate of *S. japonicum* in sentinel mice in Yunnan province with a random-effects analysis.

**Fig S4.7** Forest plot of infection rate of *S. japonicum* in sentinel mice in Sichuan province with a random-effects analysis.


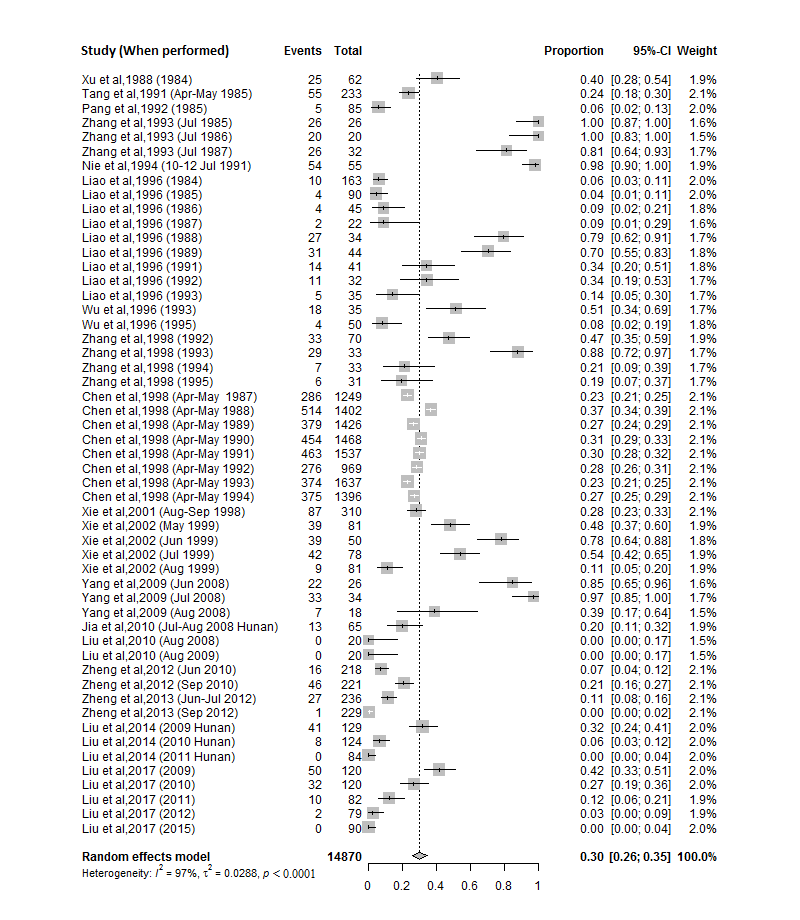


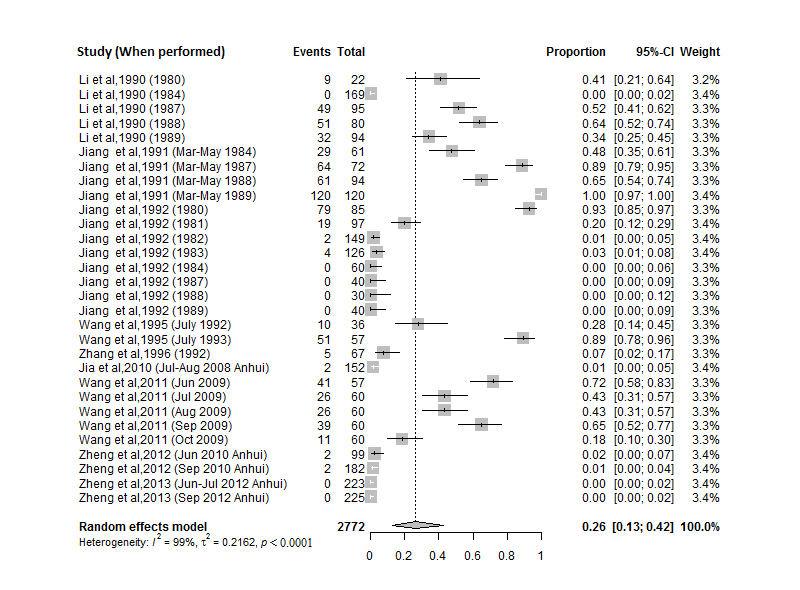


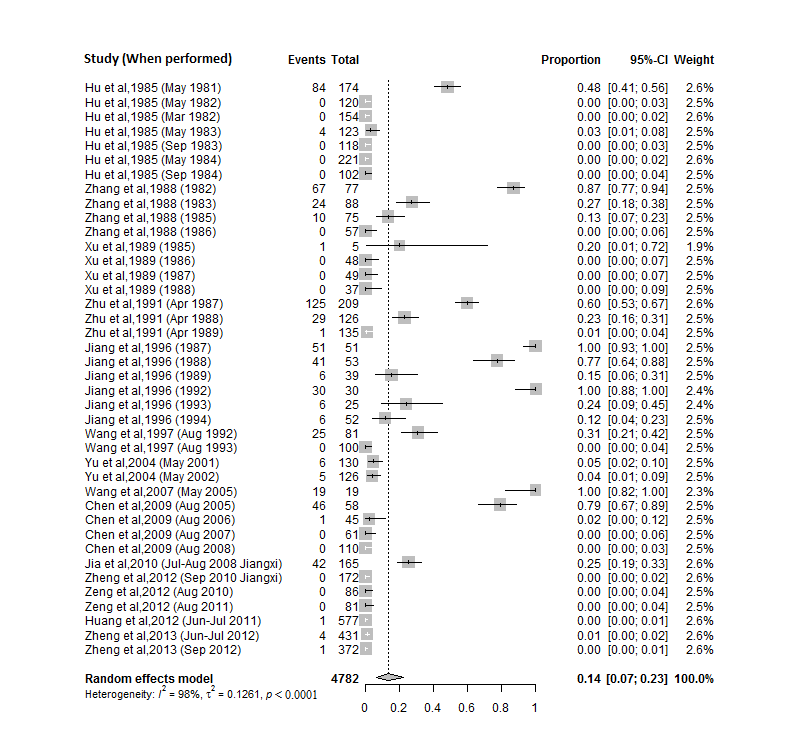


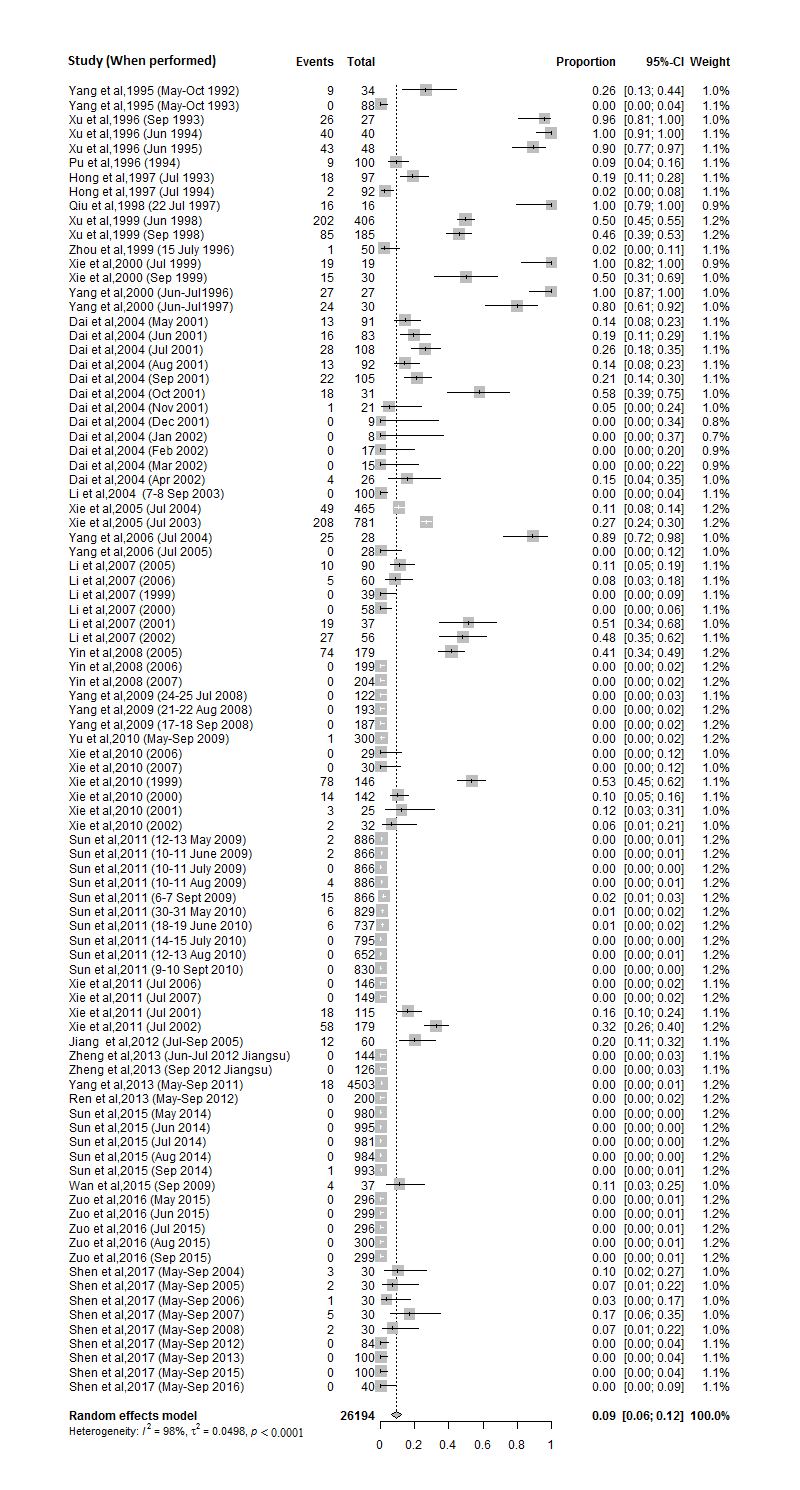


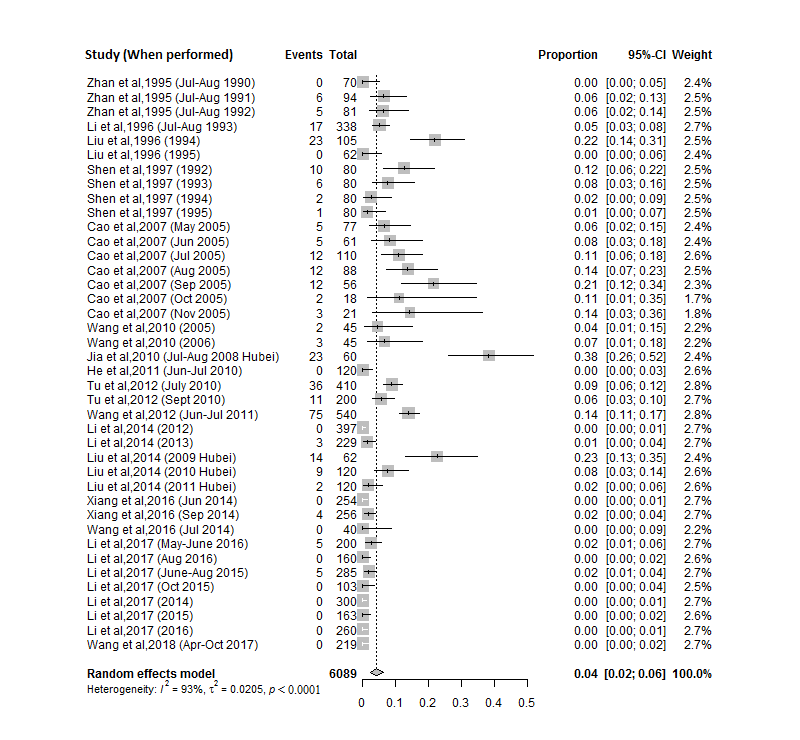


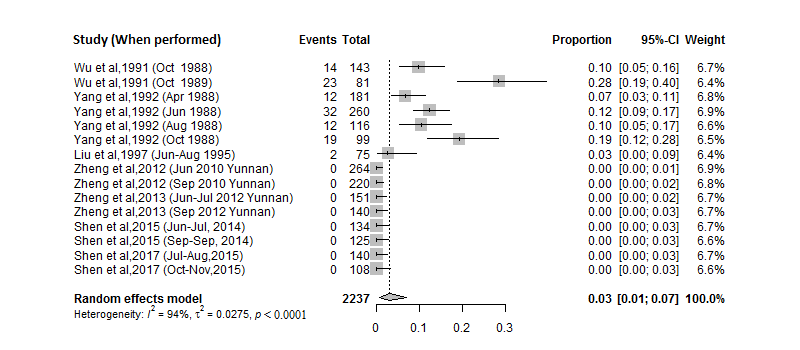


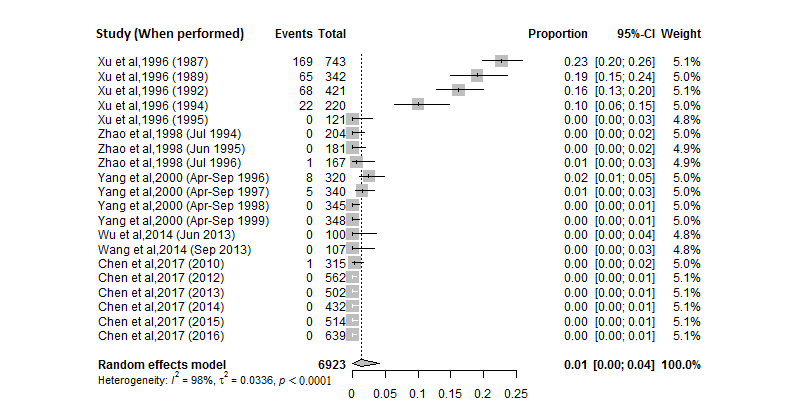

Supplement: S4 Fig — (DOC) [file pntd.0007475.s007.doc]
